# Exosomal TNF-α mediates voltage-gated Na^+^ channel 1.6 overexpression and contributes to brain tumor–induced neuronal hyperexcitability

**DOI:** 10.1172/JCI166271

**Published:** 2024-08-01

**Authors:** Cesar Adolfo Sanchez Trivino, Renza Spelat, Federica Spada, Camilla D’Angelo, Ivana Manini, Irene Giulia Rolle, Tamara Ius, Pietro Parisse, Anna Menini, Daniela Cesselli, Miran Skrap, Fabrizia Cesca, Vincent Torre

**Affiliations:** 1 International School for Advanced Studies (SISSA), Trieste, Italy.; 2 Institute of Materials (IOM-CNR), Area Science Park, Basovizza, Trieste, Italy.; 3 Department of Life Sciences, University of Trieste, Trieste, Italy.; 4 Department of Medicine, University of Udine, Udine, Italy.; 5 Institute of Pathology and; 6 Neurosurgery Unit, Department of Neurosciences, Santa Maria della Misericordia University Hospital, Udine, Italy.; 7 BISS GlioGuard Srl, Trieste, Italy.; 8 Suzhou Institute of Nano-Tech and Nano-Bionics, Chinese Academy of Sciences, Jiangsu, China.

**Keywords:** Neuroscience, Oncology, Brain cancer, Epilepsy, Sodium channels

## Abstract

Patients affected by glioma frequently experience epileptic discharges; however, the causes of brain tumor–related epilepsy (BTRE) are still not completely understood. We investigated the mechanisms underlying BTRE by analyzing the effects of exosomes released by U87 glioma cells and by patient-derived glioma cells. Rat hippocampal neurons incubated for 24 hours with these exosomes exhibited increased spontaneous firing, while their resting membrane potential shifted positively by 10–15 mV. Voltage clamp recordings demonstrated that the activation of the Na^+^ current shifted toward more hyperpolarized voltages by 10–15 mV. To understand the factors inducing hyperexcitability, we focused on exosomal cytokines. Western blot and ELISAs showed that TNF-α was present inside glioma-derived exosomes. Remarkably, incubation with TNF-α fully mimicked the phenotype induced by exosomes, with neurons firing continuously, while their resting membrane potential shifted positively. Real-time PCR revealed that both exosomes and TNF-α induced overexpression of the voltage-gated Na^+^ channel Nav1.6, a low-threshold Na^+^ channel responsible for hyperexcitability. When neurons were preincubated with infliximab, a specific TNF-α inhibitor, the hyperexcitability induced by exosomes and TNF-α was drastically reduced. We propose that infliximab, an FDA-approved drug to treat rheumatoid arthritis, could ameliorate the conditions of glioma patients with BTRE.

## Abstract 2

We describe that tumor necrosis factor (TNF) alpha released by patient-derived tumor exosomes contributes to the onset of brain tumor-related epilepsy (BTRE).

## Introduction

Patients with brain tumors develop symptoms ranging from headaches to epileptic discharges to impairment of specific cognitive functions (1–3). The mechanisms leading to brain tumor–related epilepsy (BTRE) are not completely understood, and it is thought to have multiple origins (4, 5). The main causes of BTRE are ascribed to tumor growth, disruption of the blood-brain barrier (BBB), altered synaptic functions, and pathologies of the communication between neurons and other brain cells. BTRE is certainly caused by the growth of the tumor and the associated unusual pressure on the healthy tissue but can also be caused by the release of specific factors from tumor cells. Gliomas release several factors in the microenvironment; among these factors are extracellular vesicles (EVs), including exosomes (6, 7), which contain a large variety of molecules, including proteins — in particular, cytokines — and small RNAs (8–13). EVs are released by all cells and regulate intercellular communication in both health and disease conditions (14). EVs thus play a crucial role in tumor growth, invasion, metastasis, angiogenesis, and immunity and mediate critical communication between the tumor cells and their microenvironment to sustain the glioma. Glioma-derived EVs deliver unique cargoes such as proteins, nucleic acids, and lipids to recipient cells, to alter their gene expression profile and phenotypes (15).

Cancer stem cells in glioma have been described by several studies (16–18). Glioma stem cells (GSCs) are shielded in a particular niche, where factors are released in order to maintain their state (19–22). Glioma-associated stem cells (GASCs) are a population of stem cells representative of the tumor microenvironment. GASCs are present in gliomas, and, although not tumorigenic, they support the aggressiveness of GSCs. Moreover, GASCs influence different processes, such as tumor progression, cell deformability, and interactions with GSCs (23, 24). The release of exosomes contributes to the tumor-supporting features of GASCs, increasing proliferation, motility, and anchorage-independent growth of GSCs (23). Furthermore, SEMA7A carried by GASC-derived exosomes enhances the motility of GSCs, via interaction with β_1_-integrin, expressed on GSCs’ surface (25). We recently showed that exosomes released from GASCs induce a massive crosstalk between tumor and neurons, inducing a global alteration of network activity (26).

Here, we studied the effect of glioma-derived exosomes on the firing properties of primary rodent neurons, with the aim of gaining more insights into the molecular mechanisms that induce BTRE. We used exosomes secreted by U87 cells and by glioma-derived cells obtained from patients who underwent surgery. Our findings can be summarized as follows: (a) Both cells’ and patients’ exosomes induced an increase of spontaneous firing. (b) Exosomes contain the cytokine tumor necrosis factor-α (TNF-α), which induces an almost steady and spontaneous firing in healthy cerebral neurons (27–29). (c) A detailed analysis of the biophysical properties of the voltage-gated currents revealed that both exosomes and TNF-α shift the activation of Na^+^ currents toward more negative voltages and, as a consequence, neurons are hyperexcitable and fire continuously. (d) Infliximab, an FDA-approved drug (30), reduces the heightened firing induced in vitro by exosomes and by TNF-α. This result can be applicable in the clinical setting by bridging the knowhow of neurosurgeons with that of neurobiologists. Infliximab is expected to ameliorate the conditions of patients with glioma experiencing epileptic seizures.

## Results

### U87 and patients’ exosomes induce increased spontaneous firing in primary neurons.

Exosomes were isolated from the conditioned medium of patient-derived GSCs and GASCs (23, 25, 31) and characterized by nanoparticle tracking analysis to define size and concentration. To verify the identity of isolated particles, the expression of specific exosomal markers such as Flotillin1, tumor susceptibility gene 101 (TSG-101), and programmed cell death 6–interacting protein (ALIX) was assessed (26). Atomic force microscopy was used to visualize isolated exosomes, while in the control samples only smaller particles were identified, probably residues of the isolation procedure (Supplemental Figure 1A; supplemental material available online with this article; https://doi.org/10.1172/JCI166271DS1). Isolated vesicles revealed a diameter of about 130 nm (Supplemental Figure 1B), as expected for small/medium extracellular vesicles (23, 32, 33).

Dissociated hippocampal neurons at days in vitro (DIV) 8–15 (in a few experiments also cortical neurons, as detailed) were incubated for 24 hours with 4.2 × 10^3^ U87 exosomes per neuron, and the electrical properties before and after incubation were compared. No estimation of exosome concentration in the peritumoral tissue is available in the literature, likely because of the many variables involved. For our experiments we used exosome concentrations of 30 μg/mL, in line with ref. 34 (1–100 μg/mL) and references therein, i.e., ref. 35 (30 μg/mL) and ref. 36 (50 μg/mL), and lower than what was used in primary MN cultures (37), i.e., 5 × 10^5^ particles per cell (our concentration: 2.1 × 10^3^ to 4.2 × 10^3^ particles per cell), indicating that we were operating within a low to medium range of exosome concentration.

**Figure 1:**
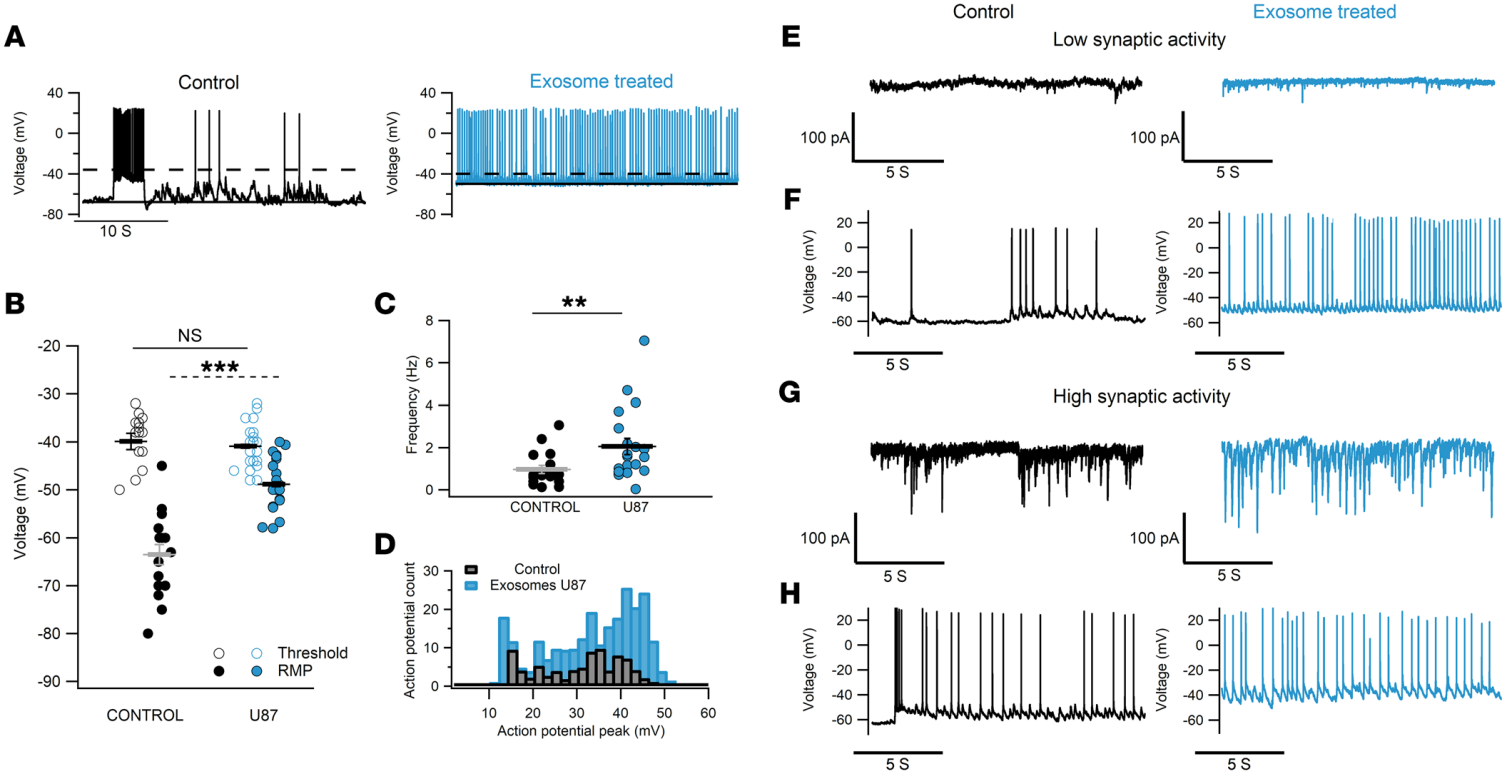
U87 exosomes induce increased spontaneous firing that is independent of the synaptic input. (**A**) Intracellular recordings in current clamp (I = 0) from a control hippocampal neuron (left, black trace) and a neuron incubated for 24 hours with U87 exosomes (right, blue trace). (**B**) Comparison of resting membrane potential (RMP; filled circles) and AP threshold (open circles). (**C**) Spontaneous AP frequency for both conditions. For **B** and **C**, *n* = 16 control, *n* = 20–22 treated neurons. **P < 0.01, ***P < 0.001, Mann-Whitney *U* test. (**D**) AP distribution from 8 randomly selected neurons in control and treated groups. (**E**) Neurons exhibiting low synaptic inputs: representative examples of voltage clamp recordings at –70 mV under control and treated conditions to obtain recordings of synaptic currents not contaminated by voltage-gated conductances. (**F**) Corresponding current clamp recordings from the cells in **E**, showing increased firing induced by U87 exosomes (blue). (**G** and **H**) Voltage clamp at –70 mV (**G**) and current clamp (**H**) obtained from a control and a U87-treated neuron exhibiting high synaptic inputs.

**Figure 2:**
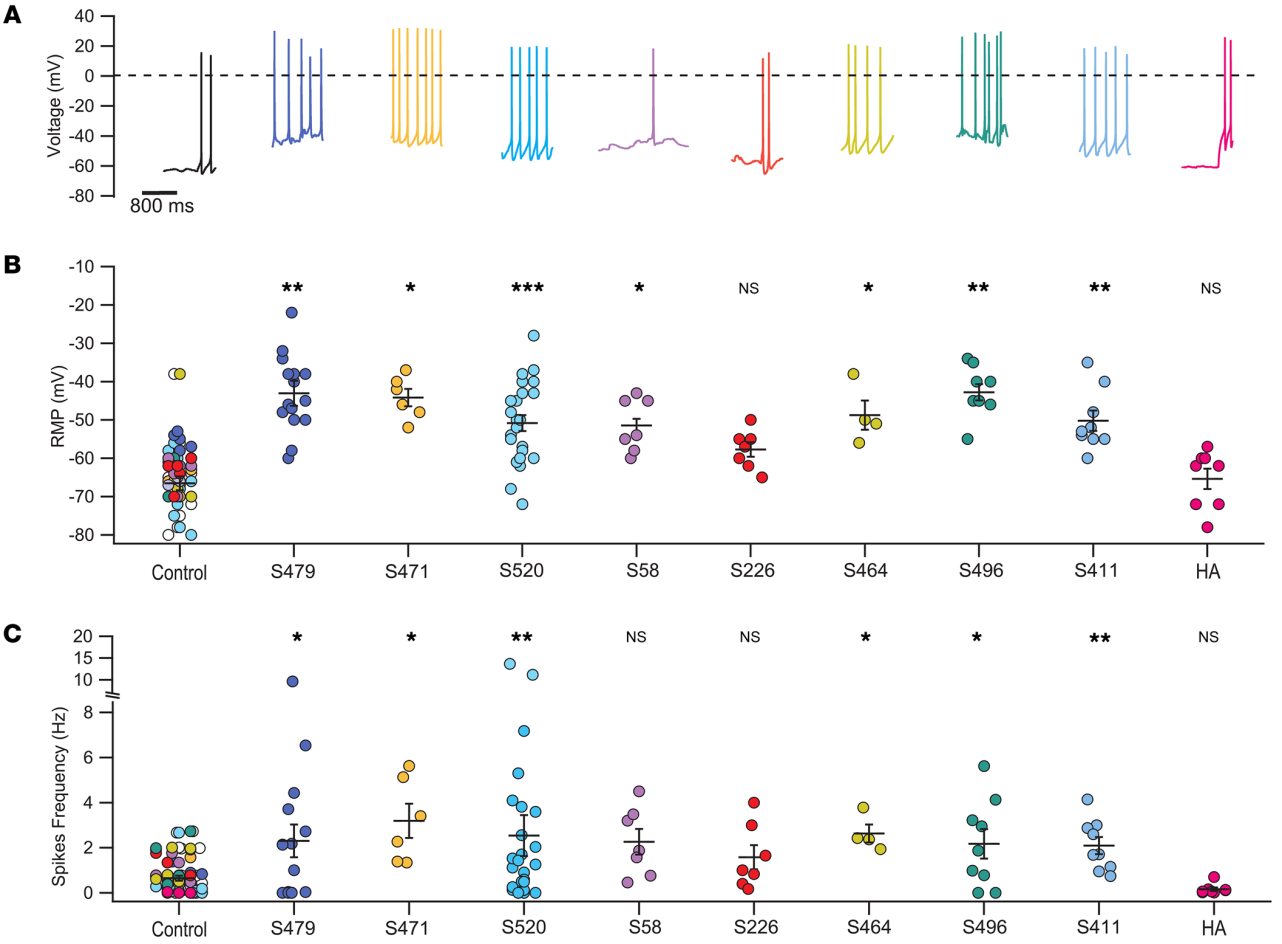
The increase of spontaneous firing is also induced by patient-derived exosomes. (**A**) Representative current clamp recordings from control hippocampal neurons (leftmost black trace) and neurons incubated with patients’ exosomes (colored traces) and with exosomes obtained from healthy human astrocytes (HAs; rightmost pink trace); the dashed black line shows 0 mV. Patients’ identification numbers are indicated. (**B**) RMP for all the experimental groups. Values for exosomes derived from patients with an epileptic report were –43 ± 2.6 mV for GASC-S479 and –42.8 ± 2.13 for GSC-S496, which were significantly different from the values of their control groups, i.e., –61.7 ± 4.4 mV and –76.8 ± 7.3 mV, respectively. (**C**) Spontaneous firing frequency of the same experimental groups: 2.3 ± 0.73 Hz for GASC-S479, 2.17 ± 0.65 Hz for GSC-S496; the respective controls were 0.56 ± 0.14 Hz and 1.46 ± 0.54 Hz. Data from control neurons for all the experimental groups are overimposed in the leftmost column. *P < 0.05, **P < 0.01, ***P < 0.001, Mann-Whitney *U* test, *n* = 4–25.

In all experiments, control samples were treated with nanoparticles obtained from culture medium that did not come into contact with cells, subjected to the same exosome isolation procedure. Additional control groups were performed in some experiments and are described where pertinent. Current clamp recordings of control neurons (black traces and symbols in Figure 1, A–C) showed a resting membrane potential (RMP) of –63.5 ± 2 mV (*n* = 16), action potential (AP) threshold at –40 ± 1.4 mV (*n* = 16), and a firing pattern in bursts with a mean AP frequency of 0.98 ± 0.2 Hz (38–40). In contrast, neurons incubated with U87 exosomes had a more depolarized RMP of –48.8 ± 1.15 mV (*n* = 22) and fired spontaneously (2.05 ± 0.38 Hz; blue traces and symbols in Figure 1, A–C). Increased excitability was not seen following incubation with exosomes deriving from healthy human astrocytes (HAs), an additional control group (Figure 2). To investigate whether exosome exposure had any adverse effect on neurons, resulting in an injury discharge and a consequent increased firing, we computed the distribution of AP voltage peak in control and treated neurons and found that 90% of the values were distributed in the range between +10 and +50 mV (Figure 1D). In addition, the mean values of the AP peak in both experimental groups were comparable (treated neurons 32.7 ± 1 mV and control 33.1 ± 0.94 mV; Supplemental Figure 2). Current clamp recordings show that control neurons exhibited spontaneous bursts of APs and had clear synaptic input. Neurons firing more vigorously also had larger synaptic currents, as evidenced by the voltage clamp recordings at –70 mV (Figure 1, E–G, black traces) and as previously reported (41–45). Conversely, the spontaneous firing of neurons preincubated with exosomes was almost independent of the amount and frequency of synaptic currents; indeed, the higher AP activity of treated neurons was observed both in the presence and in the absence of strong synaptic inputs (Figure 1, E–H, blue traces).

We performed similar experiments with GSC and GASC exosomes from 8 patients (Supplemental Table 1). Rodent hippocampal neurons were treated with these exosomes, at the same concentration as in previous experiments. Collected data show that the average RMP of neurons treated with patients’ exosomes varied between –42.8 (patient S496) and –57.7 (patient S226) mV, more depolarized than untreated neurons, whose average values varied between –59.2 and –76.8 mV (Figure 2, A and B, and Supplemental Table 2). Moreover, treated neurons had a more vigorous spontaneous activity, with the exception of patients S58 and S226. The firing frequency (Figure 2, A and C) of treated neurons showed average values between 3.19 (S471) and 1.58 Hz (S226), higher than that of control neurons (1.46 to 0.13 Hz; Supplemental Table 3). The effect of exosomes from different patients was variable, and we could not make a correlation with their clinical history, which was not available to us. However, we had access to a short clinical summary, and we noted that exosomes from patients with severe epileptic episodes (GSC-S496 and GASC-S479) induced a more depolarized shift of the RMP and more intense firing. The RMP and the firing frequency of neurons treated with exosomes from HAs (Figure 2, pink traces and symbols) were –65.4 ± 2.7 mV and 0.16 ± 0.08 Hz, respectively, similar to those of control neurons. Description of the statistical significance for different patients is reported in the legend to Figure 2. For some patients we had a limited amount of material, sufficient for few experiments. However, even samples with low numerosity showed low variability, and in addition, data obtained with exosomes derived from different patients are consistent, supporting our conclusions. The effect of exosomes on cortical neurons was more variable than their effect on hippocampal neurons (Supplemental Figure 3), possibly because of the greater heterogeneity of cortical neurons (46).

### The effect of patient-derived exosomes depends on the brain region from which they are derived.

**Figure 3:**
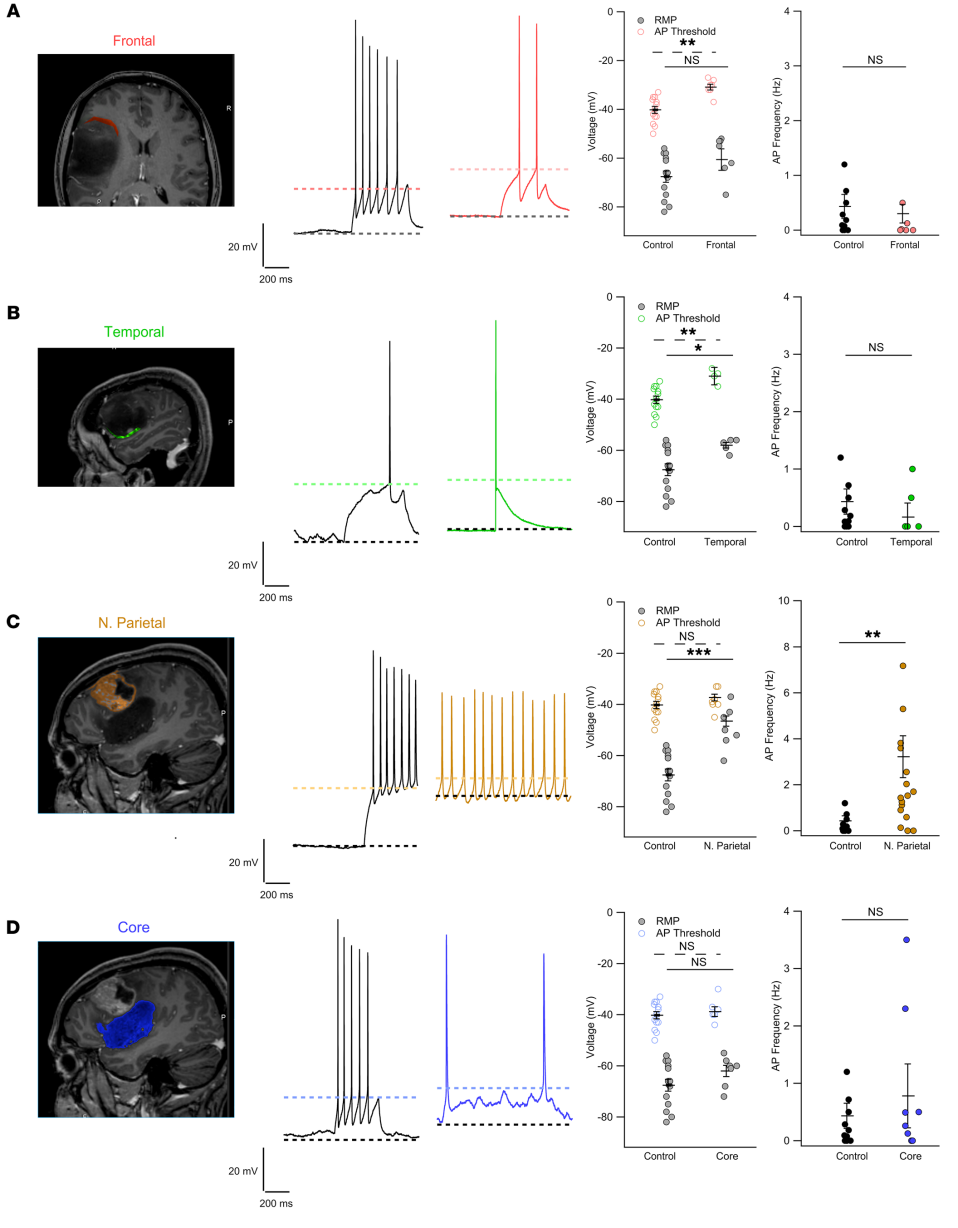
The effect of patient exosomes depends on the region they derived from. Panels show, from left to right: tomography images of patient S520 highlighting the different portions of the tumor; traces from control neurons (black) and from neurons treated with exosomes derived from the different tumor areas (colored traces); and quantification of RMP, AP threshold, and AP frequency. (**A**) Coronal tomography section showing the tumoral region in close contact with the frontoparietal lobe highlighted in red. RMP: –67.57 ± 3.31 mV (control) and –59.1 ± 3.2 mV (exosomes); AP threshold: –40 ± 1.4 mV (control) and –30.8 ± 1.24 mV (exosomes); spike frequency: 0.43 ± 0.22 mV (control) and 0.09 ± 0.07 Hz (exosomes). (**B**) Sagittal tomography section showing the temporal border of the tumor highlighted in green. RMP: –58 ± 1.14 mV; AP threshold: –31 ± 1.5 mV; spike frequency: 0.3 ± 0.2 Hz. Control values as in **A**. (**C**) Sagittal tomography section showing a tumor nodule localized in the parietal lobe highlighted in yellow. RMP: –46.5 ± 1.95 mV; AP threshold: –37.3 ± 1.26 mV; spike frequency; 3.22 ± 0.9 Hz. Control values as in **A**. (**D**) Sagittal tomography section showing the tumor mass localized in the temporal lobe highlighted in blue; exosomes were extracted from the core of this mass. RMP: –62 ± 2.23 mV; AP threshold: –38.2 ± 2 mV; spike frequency: 0.9 ± 0.45 Hz. Control values as in **A**. Kruskal-Wallis followed by Bonferroni-corrected Dunn’s test for all groups vs. control. *P < 0.05, **P < 0.01, ***P < 0.001; control *n* = 14 (common to all experimental samples), temporal *n* = 4–5, frontal *n* = 7, parietal *n* = 18, core *n* = 6–8.

To better examine the impact of patient exosomes, we investigated whether the observed results could be influenced by the brain region from which they originated. We collaborated with neurosurgeons and neurologists performing electroencephalographic measurements during surgery. During a surgery of a brain solid tumor, 4 samples from different brain areas were obtained (Figure 3, A–D). These fragments were dissociated, GSCs were cultured, and secreted exosomes were harvested. Exosomes from a fragment classified as a grade IV malignant glioblastoma induced heightened firing in hippocampal neurons, with increased firing rate (Figure 3C). In contrast, exosomes from GSCs from low-grade fragments did not elicit such behavior (Figure 3, A, B, and D). Notably, we observed a trend shift in the RMP toward more positive values, which reached statistical significance for the temporal and parietal fragments, in agreement with our previous data. These results suggest that the effect of exosomes depends on both the cortical area from which they derived and the degree of the tumor, consistent with our previous observations (47), highlighting the complexity of BTRE and of exosome action.

### Exosomes increase neuronal excitability independently of the cellular type, accelerating the depolarizing phase of AP initiation.

We then investigated whether the exosome effects differed depending on hippocampal neuron morphology (48). We loaded the solution filling the patch electrodes with fluorescein (49) to distinguish neurons with a bipolar or pyramidal morphology (50–55) (Supplemental Figure 4, A and B). The results evidenced that both bipolar and pyramidal neurons treated with exosomes increased their spontaneous firing and had a more depolarized RMP (Supplemental Figure 4B).

Then, we plotted the spontaneous firing activity measured in current clamp (I = 0) against the synaptic input (measured in voltage clamp at –80 mV) for each cell, searching for any evidence of coupling between the activity of these neurons within the network (Supplemental Figure 4, C and D). As expected, control neurons exhibited a positive correlation with synaptic input (*r*^2^ = 0.72). In contrast, in exosome-treated neurons AP firing became independent of synaptic activity (Supplemental Figure 4D). Next, we compared pyramidal neurons with high spontaneous activity (Supplemental Figure 4E), quantifying their GABAergic and glutamatergic events, based on their characteristic decay time constants. We found that AP firing in these cells was independent of GABA input (Supplemental Figure 4, F and G), with no evident correlation with GABA input frequency (Supplemental Figure 4H). This suggests that exosomes might disproportionately affect GABAergic cells, rendering their synaptic input silent and incapable of counteracting the increased excitability of pyramidal neurons.

**Figure 4:**
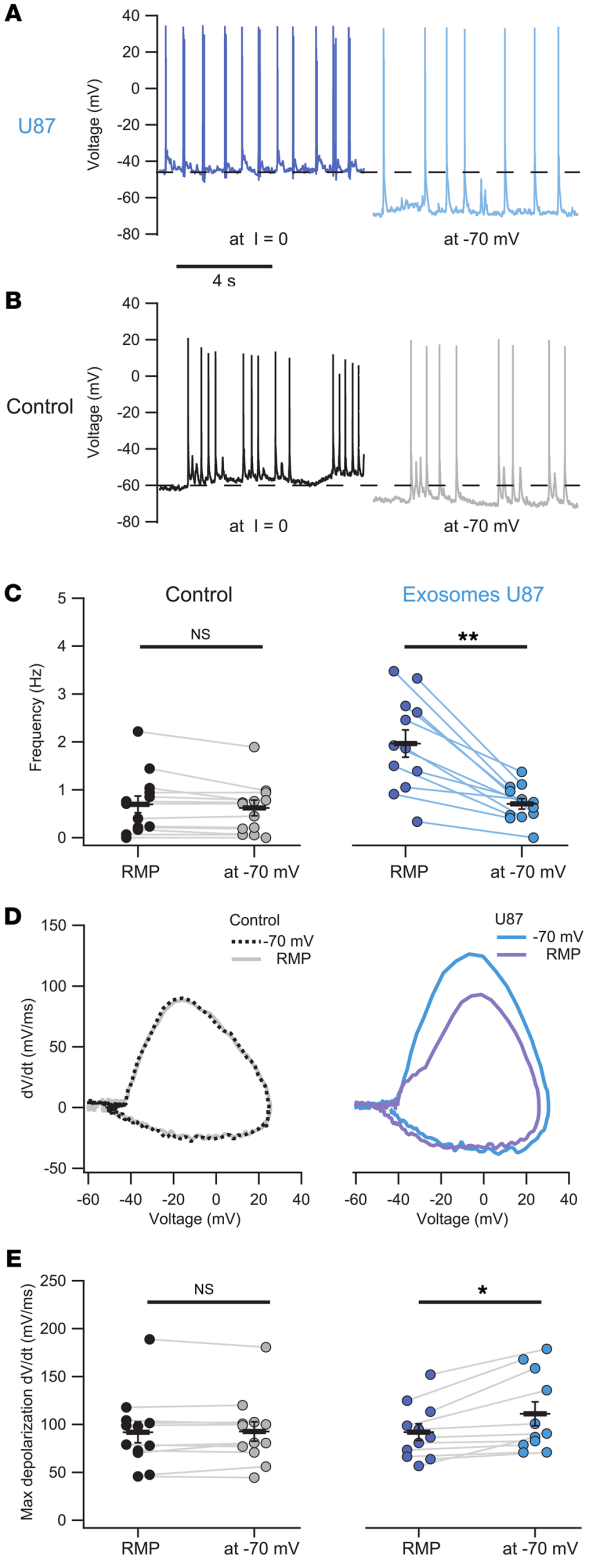
Exosomes increase excitability of hippocampal neurons, accelerating the depolarizing phase of AP initiation. (**A**) Representative traces of treated neurons recorded in current clamp (I = 0) display a highly depolarized RMP (dark blue) and the same cell held at –70 mV. (**B**) Control neurons recorded as in **A**. (**C**) Comparison of the spontaneous activity frequency of hippocampal neurons held at RMP (I = 0) versus –70 mV. Control neurons had an average frequency of 0.69 ± 0.18 Hz at RMP, with no significant change observed when hyperpolarized to –70 mV (0.62 ± 0.16 Hz; *P* > 0.05, Wilcoxon’s paired samples test). In contrast, treated neurons decreased their spontaneous activity when a hyperpolarizing current was injected, reducing the frequency from 1.96 ± 0.28 Hz at RMP to 0.7 ± 0.11 Hz at –70 mV (**P < 0.01, Wilcoxon’s paired samples test). (**D** and **E**) Mean AP phase plot shows increased maximum depolarization rate (dV/dt) when U87 exosome–treated neurons were held at –70 mV (111.1 ± 12.4 mV/ms) compared with those at RMP (92 ± 8.7 mV/ms; *P < 0.05, Wilcoxon’s paired samples test). This effect was not observed in control neurons, which exhibited similar rates both at –70 mV (92.7 ± 10.08 mV/ms) and at RMP (91.8 ± 11 mV/ms; *P* > 0.05, Wilcoxon’s paired samples test). In all panels, *n* = 12 control, *n* = 13 treated.

We also verified that neurons treated with U87 exosomes, when injected with hyperpolarizing currents (–10 to –70 pA) bringing their RMP close to –70 mV, i.e., approximately the value of control neurons, decreased their firing frequency (Figure 4, A and C, dark/light blue traces and symbols), which approached that of untreated neurons. Notably, the same procedure did not elicit any change in frequency in control neurons (Figure 4, B and C, black/gray traces and symbols). Moreover, the analysis of the AP phase plot, i.e., dV/dt versus V, revealed that treated neurons held at –70 mV exhibited a significant increase in the maximum depolarization rate (dV/dt), as compared with control neurons (Figure 4, D and E, dark/light blue traces and symbols). In contrast, the control group did not display significant changes of the dV/dt versus V plot when the cells were held at –70 mV (Figure 4, D and E, black/gray traces and symbols).

### Concentration-dependent effect of exosome treatment.

We incubated hippocampal neurons with exosomes from patient S479 at 2 concentrations: that used for all previous experiments (4.2 × 10^3^ particles per neuron) and half (2.1 × 10^3^ particles per neuron). Under these conditions, the RMP progressively shifted to more depolarized values, i.e., –46.5 ± 4.2 mV for the low concentration and –37.3 ± 1.76 mV for the high concentration, as compared with the control value of –57 ± 2.34 mV (gray and light blue traces and points, Supplemental Figure 5, A and B). Subsequently, in voltage clamp experiments, we determined the initial voltage evoking an inward current (FV-IC), which also showed a concentration-dependent shift toward more negative values: –42.2 ± 1.8 mV for the low concentration and –55.3 ± 0.66 mV for the high concentration, in comparison with control neurons (–37.6 ± 2.1 mV).

**Figure 5:**
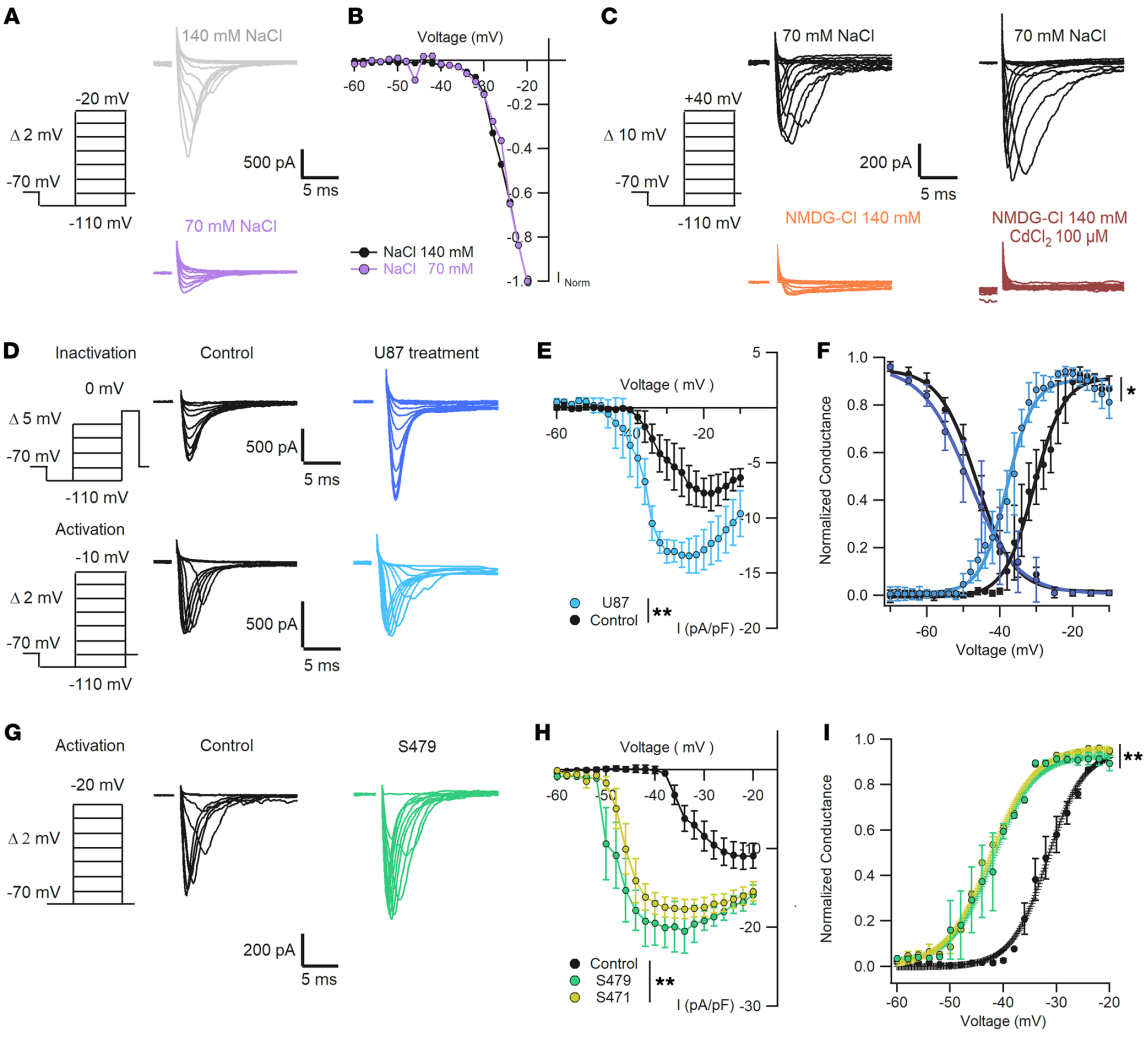
Increased spontaneous firing is associated with a shift of the activation curve of the inward voltage-dependent Na^+^ current. (**A**) Representative traces of voltage-dependent Na^+^ current activation in the presence of 140 mM (gray) and 70 mM (purple) NaCl. (**B**) Normalized currents in **A** show similar voltage activation dependence. Kruskal-Wallis test, *P* > 0.05, *n* = 5. (**C**) Replacement of NaCl by NMDG-Cl shows a residual small inward current (orange) blocked by addition of CdCl_2_ 100 μM (brown); *n* = 5. (**D**) Representative traces of voltage-dependent Na^+^ currents obtained with CdCl 100 μM in the extracellular solution and internal solution with CsCl 135 mM plus NaCl 5 mM. Currents were recorded with the protocols used for activation and inactivation in control (black: *n* = 7–10) and U87 exosome–treated (blue: *n* = 12–14) neurons. (**E**) I-V curve comparing current density as in **D**, showing increased current in treated neurons (Kruskal-Wallis test, **P < 0.01). (**F**) Dependence of normalized conductance G/max G as a function of voltage for control and treated neurons, showing an average V_1/2_ shift of –6.4 mV (values in main text; *P < 0.05, Mann-Whitney *U* test). (**G**) Representative traces of Na^+^ currents activated under control conditions (black, *n* = 7) and in neurons treated for 24 hours with exosomes from patient S479 (green, *n* = 7). (**H**) Average I-V curves of Na^+^ currents for control and patient exosome–treated neurons, showing higher Na^+^ current density in cells treated with patients’ exosomes (**P < 0.01, Kruskal-Wallis test). (**I**) Normalized conductance curve for the conditions in **H** showing early activation of the Na^+^ conductance of neurons treated with patients’ exosomes (V_1/2_ shift of –12 ± 7.2 mV, values in main text; **P < 0.01, Mann-Whitney *U* test). Voltage clamp protocols to test Na^+^ current activation and inactivation are shown on the left of each panel.

In treated neurons (both low and high concentration), FV-IC almost coincided with the RMP of the same cells, consistent with the observed increase of spontaneous AP frequency. For the high-concentration neurons, the FV-IC was more hyperpolarized than the RMP, and neurons either fired almost continuously (pink traces in Supplemental Figure 5A) or were unable to generate spontaneous AP, most likely because of the inactivation of the inward current (Supplemental Figure 6A), as further shown in Figure 5. However, hyperpolarization of the same cells induced firing, verifying the increase in intrinsic excitability (Supplemental Figure 6, B and C). These findings indicate that the effect of exosomes is concentration dependent and that exposure to exosomes primarily modifies the voltage-gated conductance responsible for spike initiation.

### Voltage-gated sodium currents are altered by patient-derived exosomes.

In order to collect voltage clamp data with an acceptable spatial clamp, we impaled neurons with a small cell body and limited dendritic arborization. Given an access resistance of 3–5 MΩ, a peak inward Na^+^ current of 5 nA implies an error of 15–25 mV, which is not acceptable. If the Na^+^ current, in contrast, does not exceed 0.5–1 nA, the error is less than 3 mV, which becomes acceptable. The holding potential was –70 mV, which was moved briefly to –110 mV and then to more positive values according to the planned experiment. In the experiments aimed to determine the activation threshold of the Na^+^ current, the voltage was moved up to –20 mV in steps of 2 mV (Figure 5A). We compared data obtained with 140 and 70 mM Na^+^ in the extracellular solution (traces in Figure 5A). After normalization to the maximal recorded current, the activation curves of Na^+^ current under normal and low Na^+^ conditions were almost identical (Figure 5B), and the inward Na^+^ current became visible at voltages above –45 mV. In the experiments to establish the full range of the inward current activation, the maximum voltage was +40 mV, and in the presence of 70 mM Na^+^ in the extracellular solution, the maximal recorded current was in the range of 500 pA (black traces in Figure 5C).

The inward current responsible for the increased spontaneous firing of neurons treated with exosomes could be carried by the entry of Na^+^ and/or Ca^2+^ ions. In particular, hippocampal neurons have large voltage-gated Na^+^ currents (Nav) (53, 54). To establish the ionic identity of this current, we recorded inward currents with an intracellular solution containing Cs^+^ to block outward K^+^ currents and extracellular application of Cd^2+^ to block voltage-gated Ca^2+^ channels (Figure 5C). When Na^+^ was entirely replaced by *N*-methyl-d-glucamine (NMDG), the inward current was drastically reduced (Figure 5C, orange traces) and was completely abolished upon addition of 100 mM Cd^2+^ (Figure 5C, brown traces). These results indicate that the inward current is almost entirely carried by Na^+^ ions, but some Ca^2+^ channels are also present and are activated at positive voltages.

We then compared Na^+^ current activation and inactivation in control and treated neurons (Figure 5D) using the appropriate protocols (55, 56). The maximal amplitude of the Na^+^ current density in treated neurons was –13.4 ± 1.5 pA/pF (*n* = 14), larger than in control neurons (–7.7 ± 1.6 pA/pF; *n* = 10) (Figure 5E). The Na^+^ current of treated neurons was half activated (V_1/2_) at –37.6 ± 2 mV, about –6.4 mV more negative than that of control neurons (V_1/2_ –30 ± 1.7 mV; Figure 5, E and F). In contrast, Na^+^ current inactivation remained unaltered (–46.3 ± 1.5 mV and –48.4 ± 1.6 mV, respectively; Figure 5F). Similarly, patients’ exosomes induced a hyperpolarized shift of V_1/2_ from –32 ± 3.4 mV in control cells to –42.3 ± 4.2 mV when treated with GASC-S479 and –42 ± 3.8 mV when treated with GSC-S471 (Figure 5I). Moreover, average I-V curves of Na^+^ currents showed a higher Na^+^ current density in cells treated with patients’ exosomes compared with the control group: –10.9 ± 1.7 pA/pF for controls, –20.5 ± 2.8 pA/pF for S479, –17.7 ± 1.2 pA/pF for S471 (Figure 5, G and H). When Nav channels are activated by a strong depolarization, a small fraction, typically around 1%, do not inactivate and remain open, originating a non-inactivating persistent Na^+^ current (57). These Na^+^ channels are the substrate of the persistent current that could contribute to hyperexcitation of neurons and to epilepsy (58, 59). We attempted to isolate and compare the persistent current in control and treated neurons, applying a depolarized ramp with a rate of 0.018 mV/ms, from a holding potential of –100 mV up to –20 mV (Supplemental Figure 7A). To subtract the leakage and obtain only the persistent currents, we used the specific inhibitor riluzole (58) at 10 μM. Our experiments show the presence of a persistent current in both control and treated neurons with a significantly different activation: application of a Boltzmann equation showed a negative shift of 10.32 mV in treated neurons (Supplemental Figure 7, B and C), in agreement with the shift of the transient Na^+^ current (Figure 5).

### Exosomal TNF-α modulates neuronal Na^+^ channels.

Glioma exosomes contain a large number of proteins and small RNAs (60, 61). On the basis of previous studies, we focused on cytokines (62, 63). The most abundant cytokines in exosomes are interleukins (ILs), particularly IL-1 and IL-6, and TNF-α. IL-1β and IL-6 preferentially inhibit or decrease Na^+^ currents (64, 65), while TNF-α positively modulates Na^+^ currents in peripheral and central neurons (28, 66, 67).

**Figure 6:**
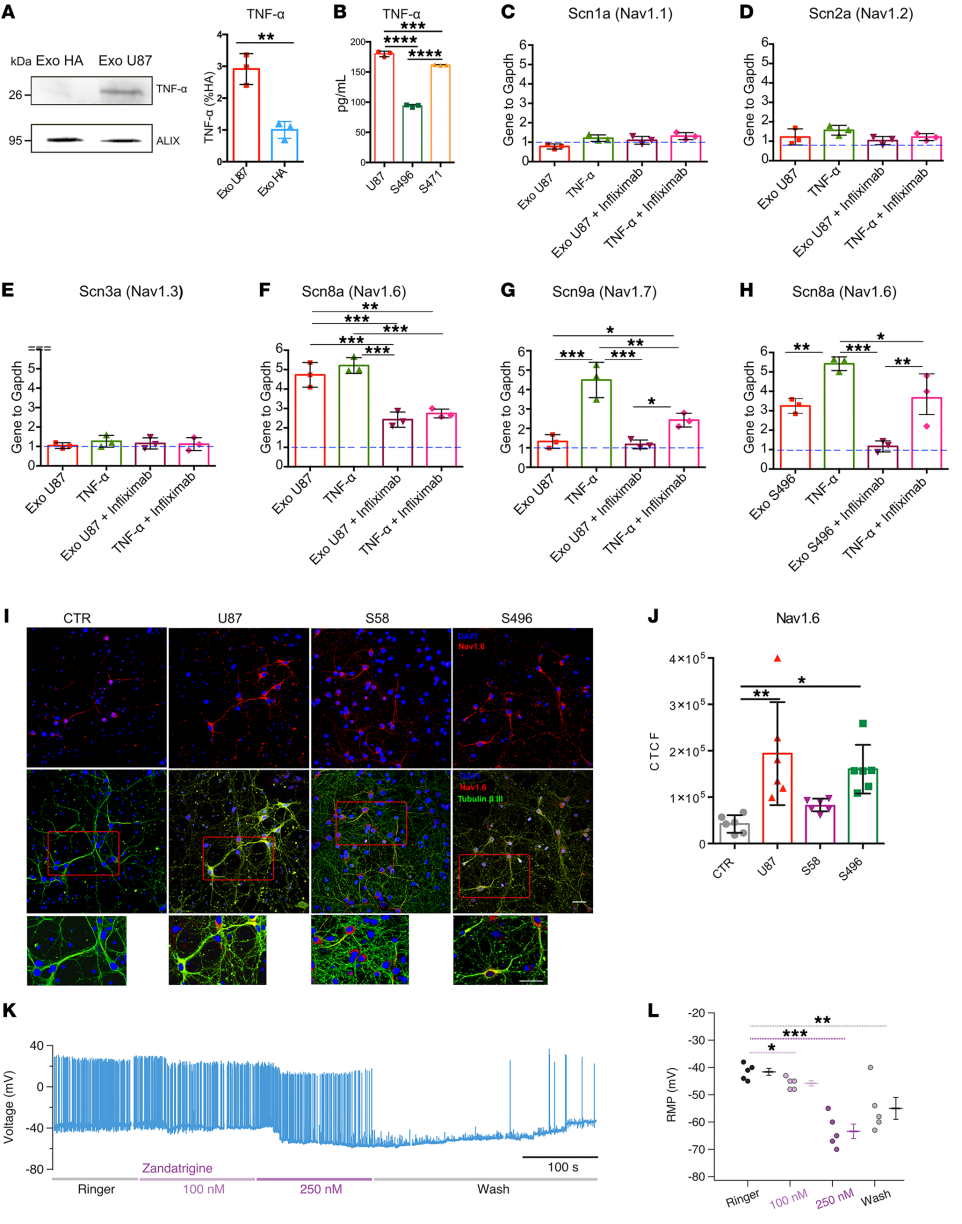
Exosomal TNF-α induces Nav1.6 overexpression. (**A**) Lysates of HA and U87 exosomes were analyzed by SDS-PAGE followed by Western blotting (left) using anti–TNF-α and ALIX antibodies. Quantification of TNF-α (right) was obtained by normalization to the exosome marker ALIX and is reported as percentage with respect to HA (*n* = 3; **P < 0.01, 2-tailed *t* test). (**B**) Quantification of TNF-α in U87 and patients S496 and S471’s exosomes using ELISA (*n* = 3 cultures). (**C**–**G**) Real-time PCR quantification of Scn1a, Scn2a, Scn3a, Scn8a, and Scn9a in hippocampal neurons treated with U87 exosomes (Exo U87), TNF-α, U87 exosomes plus infliximab pretreatment, and TNF-α plus infliximab pretreatment. (**H**) Real-time PCR quantification of Scn8a using patient S496’s exosomes. Blue dashed line represents gene expression under control conditions, set to 1. Each gene is normalized to the housekeeping Gapdh gene. *n* = 3 cultures. (**I**) Hippocampal neurons were exposed to control (CTR), U87, patient S58, and patient S496 exosomes; fixed; and stained with anti-Nav1.6 (red channel), anti–β_III_-tubulin (green channel), and DAPI to stain nuclei (blue channel). Scale bars: 50 μm. (**J**) Quantification of the experiment in **I** reported as corrected total cell fluorescence (CTCF). *n* = 6 coverslips from 2 dissections. Each point represents the average of 4 fields acquired for each coverslip. All data are shown as mean with SD. *P < 0.05, **P < 0.01, ***P < 0.001, ****P < 0.0001, 1-way ANOVA followed by Dunnett’s post hoc test. (**K**) Representative current clamp recordings from a treated neuron in the presence of increasing amounts of zandatrigine. Zandatrigine 250 nM blocked the spontaneous AP firing; this effect was partially reversible following blocker removal. (**L**) Quantification of the zandatrigine effect on RMP. *P < 0.05, **P < 0.01, ***P < 0.001, Kruskal-Wallis followed by Bonferroni-corrected Dunn’s test, *n* = 5.

We compared TNF-α expression in U87 and HA exosomes by Western blot analysis, using the exosome marker ALIX as loading control (68, 69). The results evidenced a higher expression in U87 exosomes (Figure 6A), suggesting that the increased neuronal excitability is only induced by high levels of exosomal TNF-α. We also verified the presence of TNF-α in patients’ exosomes by ELISA (Figure 6B). Next, we compared the expression of Nav channels in hippocampal neurons after 24 hours of treatment with exosomes and 10 ng/nL TNF-α by real-time PCR. TNF-α induces Nav1.6 and Nav1.7 expression in dorsal root ganglia (DRGs) (67) and in cortical neurons (66). Nav1.6 are the most abundant Na^+^ channels in the human brain and have a low threshold for spike initiation, while Nav1.2 have a much higher threshold (70). Nav1.4 and Nav1.5 are primarily expressed in muscles and heart (71) and Nav1.8 in neurons of the peripheral nervous system (72). Nav1.1 and Nav1.3 are expressed in hippocampal neurons and are involved in the induction of hyperexcitability (73, 74). Therefore, we focused on Nav1.1, Nav1.2, Nav1.3, Nav1.6, and Nav1.7 as potential candidates. Real-time PCR experiments showed that U87 exosomes induced Nav1.6 overexpression and had a negligible effect on Nav1.1, Nav1.2, Na1.3, and Nav1.7. TNF-α treatment induced a significant upregulation of Nav1.6 and Nav1.7 (Figure 6, C–G). Importantly, the increased expression of Nav1.6 was also observed in neurons treated with exosomes from patient S496 (Figure 6H). Inhibitors of TNF-α are used to treat rheumatoid arthritis and are blockers of TNFR1 and TNFR2. These inhibitors are based on a fragment, antigen binding, targeted to the binding domain of TNFR1/2 to TNF-α, and their clinical names are Remicade/infliximab, Flixabi, and Remsima (30). To verify whether TNF-α selectively enhances Nav1.6 expression, neuronal cultures were preincubated with 2.5 μg/mL infliximab for 2 hours and then treated with exosomes or TNF-α. Interestingly, pretreatment with infliximab significantly reduced Nav1.6 overexpression induced by exosomes and TNF-α (Figure 6, C and H).

To further support these results, we treated neurons with U87 and patient-derived exosomes and performed immunofluorescence experiments with anti-Nav1.6 antibodies, followed by quantitative fluorescence analysis of Nav1.6 expression (Figure 6, I and J). We observed increased Nav1.6 fluorescence intensity in neurons treated with U87 and patient S496 exosomes, consistent with the real-time PCR data. Notably, there was no increase in Nav1.6 fluorescence intensity in neurons treated with exosomes from patient S58, in line with the electrophysiological data in Figure 2. Overall, these results indicate that the increased expression of Nav1.6 is the key factor of the hyperexcitability observed in neurons treated with U87 and patients’ exosomes.

To confirm the role of Nav1.6, we investigated the effects of the specific Nav1.6 blocker zandatrigine (NBI-921352) (75). In treated neurons exhibiting a significant spontaneous firing, the progressive addition of zandatrigine hyperpolarized the RMP and abolished the spontaneous firing (Figure 6, K and L). When the blocker was removed from the extracellular medium, the RMP moved back to more depolarized values and the original spontaneous firing partially recovered. Conversely, zandatrigine had only a small effect when applied to control neurons (Supplemental Figure 8). These results further verify that TNF-α selectively enhances Nav1.6 currents.

### TNF-α mimics the effect of exosomes on primary neurons and is counteracted by infliximab.

**Figure 7:**
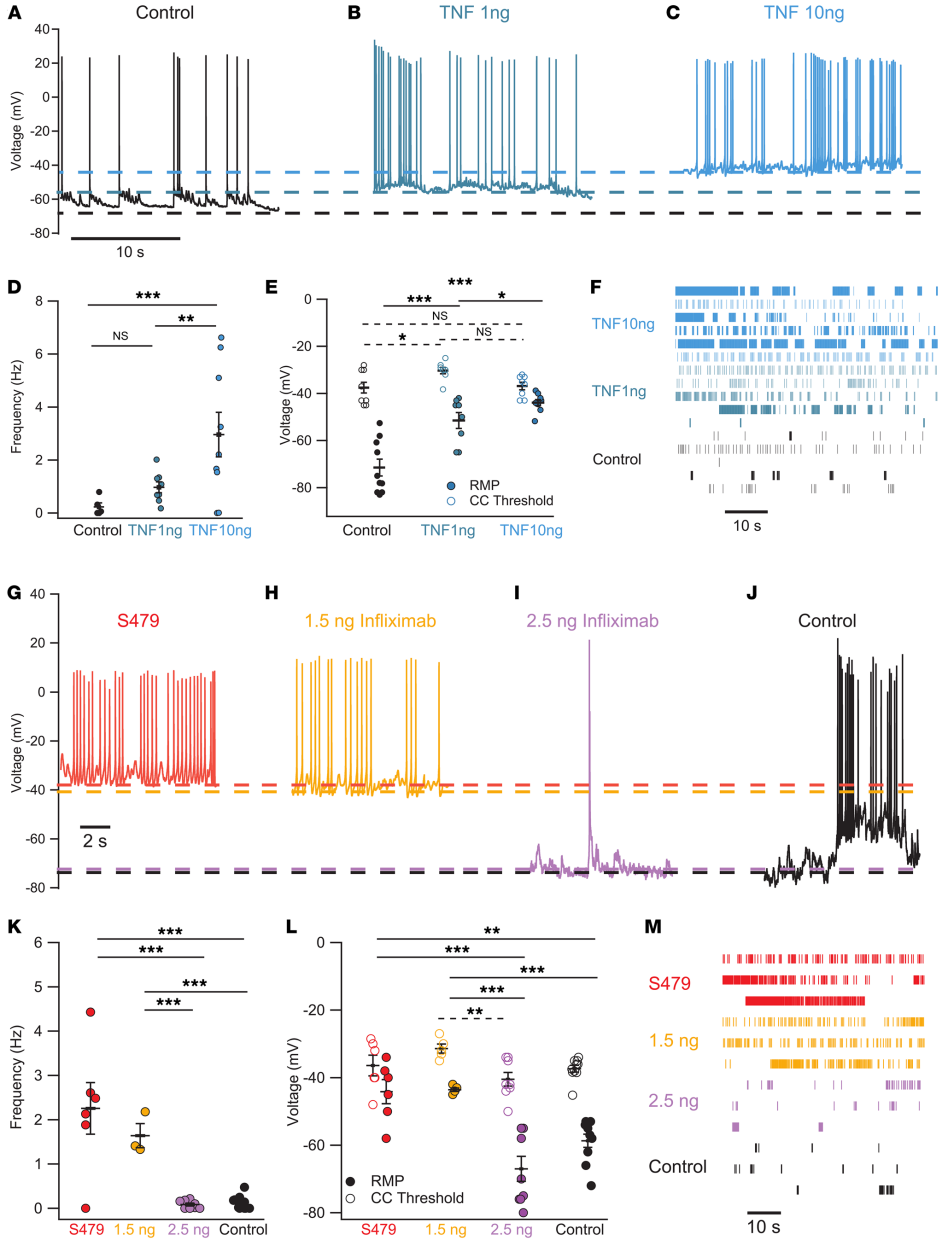
TNF-α depolarizes RMP and increases firing frequency similarly to exosomes, an effect that is antagonized by infliximab. (**A**–**C**) Representative current clamp traces under control conditions (black) and in a neuron treated for 24 hours with low (blue/green) and high (blue) TNF-α concentration. (**D** and **E**) Quantification of spontaneous firing frequency, RMP, and AP threshold for control neurons and those treated with low and high TNF-α concentration. Solid lines for RMP, dashed lines for threshold values. (**F**) Raster plots of the firing in the 3 experimental conditions. (**G** and **H**) Representative current clamp traces of a neuron treated with patient-derived exosomes (red, *n* = 6) and neurons pretreated with 1.5 ng/mL infliximab (yellow, *n* = 3–5), both showing high spiking frequency. (**I** and **J**) As in **G** and **H** but for control neurons (black, *n* = 10) and neurons pretreated with 2.5 ng/mL infliximab before exosome application (purple, *n* = 8). Dashed lines indicate AP threshold for the 4 experimental conditions. (**K**) AP frequency for the groups in **G**–**J**. (**L**) AP threshold (open circles) and RMP (filled circles) for the treatments in **G**–**J**. Infliximab 2.5 ng decreased AP frequency and increased the difference between the RMP and AP threshold. (**M**) Raster plots of the firing in the 4 experimental conditions. Control values: RMP = –58.7 ± 2 mV; threshold = –37.4 ± 1 mV. *P < 0.05, **P < 0.01, ***P < 0.001, Kruskal-Wallis followed by Bonferroni-corrected Dunn’s test.

To verify whether TNF-α elicits increased firing activity comparable to that observed with U87 and patients’ exosomes, hippocampal neurons were treated with different concentrations of TNF-α and spontaneous firing was assessed. As shown in Figure 7, A–F, incubation with 1 ng/mL TNF-α for 24 hours induced increased spontaneous firing rate (blue/green trace), which further increased when 10 ng/mL was used (blue trace). Collected data from *n* = 8 and 9 neurons showed that the mean spontaneous firing rate was 0.97 ± 0.2 Hz and 2.9 ± 0.84 Hz following incubation with 1 and 10 ng/mL TNF-α, respectively, while in control neurons values of 0.23 ± 0.15 Hz were observed (Figure 7D). As with the incubation with exosomes, the RMP approached the threshold required for spike activation: 1 ng/mL TNF-α depolarized the neurons to –51.5 ± 3.4 mV and 10 ng/mL TNF-α to –43.9 ± 1.28 mV; these values are significantly different from those of control neurons, which showed an RMP of –71.5 ± 3.6 mV (Figure 7E). TNF-α also shifted the activation curve for the inward current to more negative voltages, similarly to patients’ exosomes (Supplemental Figure 9). The effect of TNF-α and exosomes, however, was not completely identical. In fact, following incubation with 10 ng/mL TNF-α, APs showed a more pronounced undershoot of –5 ± 4.6 mV (Supplemental Figure 10A), and voltage clamp experiments showed an activation of a type A outward current with a higher amplitude in treated cells (Supplemental Figure 10, B and C), suggesting that TNF-α affected also K^+^ currents.

To further corroborate our findings, neuronal cultures were preincubated with 1.5 ng and 2.5 ng of infliximab followed by treatment with S479 exosomes (Figure 7, G–M). At the lower infliximab concentration (orange traces and symbols), neurons showed an average RMP of –43.6 ± 0.51 mV, spike threshold of –31.4 ± 1.36 mV, and AP frequency of 1.64 ± 0.27 Hz, values that were not different from those of neurons treated only with exosomes (red traces and symbols), where RMP, AP threshold, and activity frequency were –44.2 ± 3.6 mV, –36.42 ± 3.1 mV, and 2.26 ± 0.58 Hz, respectively. At the higher concentration, infliximab abolished the increased excitability induced by exosomes, as evidenced by an RMP of –68.1 ± 3.7 mV, an AP threshold of –40.5 ± 2 mV, and an AP frequency of 0.084 ± 0.032 Hz (purple traces and symbols). These values were comparable to those of control neurons (black traces and symbols) with regard to RMP (–58.7 ± 2 mV), spike threshold (–37.4 ± 1 mV), and frequency of spikes (0.13 ± 0.049 Hz). In summary, these results demonstrate that TNF-α is sufficient to trigger hyperexcitability in primary neurons. Therefore, the similar effect seen with exosomes is attributable to the presence of TNF-α.

## Discussion

In this article we propose that exosomes are important players in the origin of BTRE. We identify exosomal TNF-α as a key factor to induce neural hyperexcitability, and we propose the use of the FDA-approved drug infliximab, an inhibitor of TNF-α commonly used to treat rheumatoid arthritis (76–78), as a novel agent to treat BTRE.

### Epilepsy and brain tumors.

In BTRE the causes of epilepsy are diverse and not completely understood. We tested the effect of exosomes derived from 8 patients with glioma, and in 7 of 8 cases exosomes induced heightened firing in hippocampal neurons. This effect was specific to glioma-derived exosomes, as it was not observed with exosomes derived from human astrocytes. Importantly, exosomes derived from GSCs and GASCs give very similar results, suggesting that the mechanism under study is conserved between the 2 types of glioma cells. However, exosomes derived from different regions within or around the brain tumor have different action: we observed a general trend in the shift of RMP toward more depolarized values, which in one case resulted in a clear increase of neuronal firing frequency, and remarkably, this region was also described as the most aggressive by histochemical analysis. Indeed, the different effects observed with the various exosomal pools could also be due to the severity of the tumor (47). In addition, the removal of a section of the tumor resulted in the cessation of epileptic discharges in a nearby region. Together, our data further underline the complexity of the cellular mechanisms underlying BTRE. Indeed, some specific brain tumors, such as dysembryoplastic neuroepithelial tumors and gangliogliomas, have a high propensity to lead to epilepsy. Cortical tumors in the frontal, temporal, and parietal cortices tend to develop more frequent epileptic seizures (4). It has been suggested that permanent epileptic seizures require specific and complex changes in the microenvironment that are not possible in fast-growing tumors (79). In our experiments hyperexcitability is unlikely to be caused by an excessive level of glutamate, as it occurs in different cases of epilepsy, as we did not add any glutamate in any form to our cultures.

Thus, the causes of BTRE are diverse, and accordingly, commonly used antiepileptic drugs, such as lamotrigine, lacosamide, pregabalin, topiramate, and levetiracetam, are characterized by a wide range of mechanisms of action. Lamotrigine acts primarily as a Nav channel blocker (80), but its spectrum of action is broad. Similarly, lacosamide targets Nav channels, prolonging their inactivation, thereby limiting the maximal rate for AP firing (81). Pregabalin and gabapentinoid primarily inhibit Ca^2+^ channels (82). The molecular mechanisms of the action of topiramate and levetiracetam are less understood but could involve not only effects on Na^+^ and Ca^2+^ channels and neurotransmitter release (83), but also other not yet identified targets (84). According to our data, Nav channel blockage should effectively reduce BTRE, and indeed, we show that NBI-921352/zandatrigine, a specific blocker of Nav1.6 channels, abolishes the hyperactivity induced by exosomes and hyperpolarizes the RMP. In accordance with this idea, mutations that impair Nav1.6 functionality confer resistance to seizures (85). However, we cannot exclude that the observed effect would impact, besides epilepsy, patients’ motor/cognitive abilities, depending on the brain area involved (see Figure 3). The use of more specific inhibitors/blockers could reduce the risk of adverse effects when used within a clinical therapy.

### The content of glioblastoma exosomes and the effect of TNF-α.

Extracellular vesicles and exosomes released by U87 cells and brain tumors (13, 86) contain proteins, mRNA, and various kinds of microRNAs (87). The most abundant type of proteins present in glioblastoma exosomes are cytokines (86, 88). Cytokines are a big family of proteins comprising interleukins 1–15; growth factors; colony-stimulating factors; interferon-α, -β, and -γ; TNF; and chemokines.

TNF-α is one of the most important cytokines acting as a host defense that triggers inflammatory responses within the nervous system (89). Its signaling is initiated by TNF-α binding to 2 receptors, TNFR1 and TNFR2, differentially expressed in cells. TNF-α induces an almost steady and spontaneous firing in healthy cerebral neurons, in agreement with previous reports (27–29). Incubation with TNF-α leads to Na^+^ channel overexpression in both cortical cultures (66) and DRG neurons (90), often with a specific effect on Nav1.6 (29, 66, 91–93). In line with these findings, our results verify the overexpression of Nav1.6 and Nav1.7 channels in primary hippocampal neurons exposed to U87 and patient-derived exosomes, as well as to TNF-α. Concerning the possible underlying mechanisms, since we observe an increase in transcription of selected Nav, inhibiting protein synthesis could indeed partially block the effect of exosomes. However, an increase in membrane-targeted channels and/or changes in the kinetics of their activation/inactivation (because of phosphorylation or other posttranslational mechanisms; ref. 94) could also play a major role. We observed a significant shift of the activation curve toward more negative membrane potentials upon incubation with 10 ng/mL TNF-α (Supplemental Figure 5), while in previous reports the shift was not larger than just a few millivolts. In these reports, measured currents were on the order of some nanoamperes, such that the quality of the space clamp was not as good as when currents selected for the analysis were less than 1 nA. Nav1.6 channels are low threshold in contrast to Nav1.2 channels, which are high threshold, and in cerebral neurons Nav1.2 channels are activated at voltages 10–15 mV more depolarized than those for Nav1.6. In DRG neurons, the midpoints of activation are –24.4 and –36 mV for Nav1.2 and Nav1.6, respectively, and the threshold for activation is –57 and –70 mV for Nav1.6 and Nav1.2, respectively (95). Therefore, if TNF-α induces Nav1.6 channel overexpression, patch clamp experiments from cerebral neurons — where Nav1.2 and Nav1.6 are the most expressed Na^+^ channels — are expected to cause a shift of Na^+^ current half-activation toward hyperpolarized values.

Another important issue is the possible heterogeneous effect of Nav1.6 overexpression in different neural subtypes. Our results showed that exosomes and the subsequent activation of TNF-α induce hyperexcitability in both pyramidal and bipolar neurons, consistent with the expression of Nav1.6 in both neural subtypes (96). Additionally, this hyperexcitability is not decreased, but rather is often increased, in the presence of large synaptic inputs. The increased spontaneous activity in pyramidal neurons is largely independent of the GABAergic input, suggesting that bipolar interneurons may be uncoupled from network firing. This could be due to a disproportionate depolarizing effect on these neurons, highlighting a possible mechanism in which the glutamatergic input exceeds the inhibitory input. However, to better understand this mechanism, future work should take into account the variability within inhibitory neurons, which differ in quantity depending on the cortical area. For example, in the primary visual cortex, there are at least 13 distinct GABAergic subtypes (97).

Notably, in the present article we have not addressed the issue of whether the overexpression of Nav1.6 occurs preferentially in low- or high-grade glioma; this important aspect of BTRE will be dealt with properly in a manuscript currently in preparation.

In conclusion, we provide evidence suggesting that exosomes released by glioma trigger neuronal hyperexcitability in some patients. This action is likely to be mediated by exosomal TNF-α, which leads to Nav1.6 overexpression. Given the electrical properties of Nav1.6 channels, it is not surprising that their overexpression generates hyperexcitability. We propose that Nav1.6 overexpression is an important factor of BTRE, but certainly not the only cause, as changes of synaptic transmission and an excess of glutamate release also may play a major role. Under our experimental conditions, infliximab, an inhibitor of TNF-α, displayed a clear antiepileptogenic action. Infliximab does not cross the BBB; however, brain-penetrating forms of anti–TNF-α antibodies could be reengineered as IgG fusion proteins with a BBB molecular Trojan horse, such as the mAb against the human insulin receptor (98). In this case, infliximab is expected to ameliorate symptoms correlated to neuronal hyperexcitability, such as cognitive and motor deficits, as well as epileptic manifestations.

## Methods

### Sex as a biological variable.

Primary hippocampal neurons were obtained from P2–P3 Wistar rat pups (see below), whose sex was not identified at the time of dissection. Therefore, we did not consider sex as a biological variable in our in vitro experiments on rodent cells.

Regarding patient-derived cells, our samples derived from 6 male and 2 female patients. Sex was not considered as a biological variable in our in vitro experiments on patient-derived cells.

### Cell cultures.

Primary hippocampal neuron cultures were performed using Wistar rats (P2–P3) (Charles River). After enzymatic dissociation of hippocampal tissue with a solution containing NaCl 136.9 mM, KCl 4.9 mM, Na_2_HPO_4_ 7 mM, 4-(2-hydroxyethyl)-1-piperazineethanesulfonic acid (HEPES) 25.2 mM, NaHCO_3_ 4.2 mM, kynurenic acid 200 μM, and d(-)-2-amino-5-phosphonopentanoic acid 25 μM (all from MilliporeSigma), neurons were collected by centrifugation at 100*g* for 5 minutes and then plated on 15 mm glass coverslips (Menzel-Glaser, CB00150RA1), previously coated with poly-l-ornithine (MilliporeSigma, P4957) 0.5 μg/mL, and cultured in Neural Basal-A Medium (Thermo Fisher Scientific, 21103049) supplemented with GlutaMAX Supplement (Thermo Fisher Scientific, 35050061), gentamicin solution 10 mg/mL (MilliporeSigma, G1272), and B27 Supplement (Thermo Fisher Scientific, 17504044) at 37°C, 5% CO_2_, and 95% relative humidity.

Human U87 glioblastoma cells (MilliporeSigma, 89081402) were cultured in DMEM with GlutaMAX (Gibco, Thermo Fisher Scientific, 31966047) supplemented with 10% fetal bovine serum (FBS) (Euroclone, ECS0180L) and 1% penicillin-streptomycin (Euroclone, ECB3001D). The cultures were maintained at 37°C, 5% CO_2_, and 95% relative humidity. Medium was replaced every 3 days, and cultures were split at 70%–80% confluence.

Human astrocytes (HAs) (Thermo Fisher Scientific, N7805100) were cultured in DMEM with GlutaMAX supplemented with 10% FBS (Invitrogen, Thermo Fisher Scientific, 31966047 and ECS0180L), 1% PenStrep (100 U/mL penicillin and 100 μg/mL streptomycin; Thermo Fisher Scientific, 15070063), and N2 Supplement 100× (Thermo Fisher Scientific, 17502048) at 37°C, 5% CO_2_, and medium was replaced every 3 days. HA-derived exosomes were used as a control to verify the specificity of the effect of U87 and patients’ exosomes.

To obtain patient glioma cells, human glioblastoma samples were collected by the Neurosurgery Department of the Azienda Ospedaliera Universitaria of Udine. Briefly, tissue samples were mechanically/enzymatically dissociated, and single-cell suspensions were cultured as previously described (23).

### Exosome isolation and characterization.

Exosome isolation and characterization were performed as previously described (26). Briefly, supernatants deriving from U87, HA, and glioma cells from patients were subjected to exosome isolation using Total Exosome Isolation reagent (Invitrogen, Thermo Fisher Scientific, 4478359) following the manufacturer’s protocol. Isolated exosomes were analyzed using a 405 nm (violet) laser on a NanoSight LM10 (Malvern Panalytical) to verify their size and concentration. Hippocampal neurons were treated using 2.1 × 10^3^ particles (exosomes) per neuron (15 μg/mL) or 4.2 × 10^3^ particles (30 μg/mL) per neuron for 24 hours. For each treatment, 30 μL of PBS containing the desired amount of exosomes was added to 2 mL of culture media. For control treatment, fresh culture medium, which was never in contact with cells, was subjected to the same exosome extraction protocol and the pellet resuspended in PBS. Before electrophysiological recording, the culture medium was removed and replaced by ringer buffer.

### Atomic force microscopy imaging.

Atomic force microscopy (AFM) images were acquired with an MFP-3D Stand Alone AFM (Asylum Research) in dynamic AC mode in liquid using commercially available silicon cantilevers (Olympus Micro Cantilevers, BL-AC40TS-C2; nominal spring constant 0.09 N/m and resonant frequency 110 kHz).

For sample preparation, we incubated a freshly cleaved muscovite mica sheet (Ruby Muscovite Mica Scratch Free Grade V-1, Nanoandmore GmbH) with 20 μL of poly-l-ornithine solution 0.01% (MilliporeSigma) for 15 minutes at room temperature (RT). After gentle washing with Milli-Q water, we added a 15 μL drop of exosome suspension on the poly-ornithine–coated mica surface at RT for 15–30 minutes to allow vesicle binding through electrostatic interactions. Then we washed 5 times with PBS and imaged the sample in PBS with the AFM microscope. For each sample, 3–5 images with 5 × 5 μm or 10 × 10 μm of scan size and with a resolution of 1,024 × 1,024 pixels (pixel size ~ 10 × 10 nm) were acquired. AFM images were analyzed with Gwyddion software (http://gwyddion.net) to extract vesicle heights and diameters.

### Immunofluorescence staining.

Primary hippocampal neurons were plated at a density of 1.0 × 10^5^ on 15 mm coverslips and treated at DIV 7 as follows for 24 hours: (a) 10 ng/mL TNF-α; (b) 4.2 × 10^3^ U87 exosomes per cell; (c) 4.2 × 10^3^ patients’ exosomes per cell; (d) the previous treatments on samples pretreated for 2 hours with the TNF-α inhibitor infliximab at a concentration of 2.5 μg/mL. All the samples were compared with the negative control, i.e., the culture medium, which was never in contact with cells, subjected to the same exosome extraction procedure. After treatments, the cells were fixed with 4% paraformaldehyde for 20 minutes, washed 3 times with PBS, and incubated with glycine 1 mM for 5 minutes. After permeabilization with 0.2% Triton X-100 for 5 minutes, cells were incubated with 5% bovine serum albumin blocking solution at RT for 50 minutes. Then, coverslips were incubated with the following primary antibodies: rabbit polyclonal anti-Nav1.6/SCN8A (Abcam, ab65166; 1:200) and mouse monoclonal anti–β_III_-tubulin (Abcam, ab78078; 1:500). After 3 washes with PBS–0.01% Tween 20, the coverslips were incubated with goat anti-rabbit secondary antibodies conjugated to Alexa Fluor 594 (Invitrogen, Thermo Fisher Scientific, A11037; 1:600) and goat anti-mouse secondary antibodies conjugated to Alexa Fluor 488 (Invitrogen, Thermo Fisher Scientific, A11029; 1:600) for 1 hour at RT in a dark and wet chamber. To visualize nuclei, incubation with DAPI 0.5 mg/mL (Merck-Sigma, 32670) in PBS for 15 minutes at RT was performed. Coverslips were mounted with VECTASHIELD Vibrance Antifade Mounting Medium (Vector Laboratories, H-1700). For image acquisition, an inverted Nikon A1R confocal microscope was used. For each coverslip at least 4 fields were analyzed. *Z*-stacks were acquired using the NIS Elements Advanced Research Software (Nikon) with ×40/0.95 NA air objective and a spatial resolution of 512 pixels. Image analysis and fluorescence quantification were performed using ImageJ software (NIH). For Nav1.6 quantification, only the soma and the neurite initial segments were considered. The Simple Neurite Tracer ImageJ plug-in was applied to β_III_-tubulin green channel to select the region of interest (ROI) for each neuron. Fluorescence intensity was measured by integrated density (IntDen) and expressed as corrected total cell fluorescence corresponding to the IntDen minus the product of the area of the cell and the average of mean gray value from 3 background ROIs.

### Quantitative real-time PCR.

Quantitative real-time PCR was used to evaluate the Nav1.1, Nav1.2, Nav1.3, Nav1.6, and Nav1.7 Na^+^ channel expression. The RNeasy Mini Kit (QIAGEN, 74104) was used for isolation of total RNA from rat hippocampal neurons treated as previously described for immunofluorescence staining. After RNA extraction, a TURBO DNA-free Kit (MilliporeSigma, AM1907) was used to remove traces of genomic DNA. Then, cDNA synthesis was performed by Maxima First Strand cDNA Synthesis Kit (Thermo Fisher Scientific, K1641) and used for quantitative real-time PCR using SYBR Green Supermix (Bio-Rad, 1725270) on a CFX Connect Real-Time PCR Detection System (CFX Maestro Software). Expression levels were normalized to Gapdh, and fold change was determined by application of the 2^−ΔΔCt^ method. The following primers were used: Nav1.1: Scn1ar_fw 5′-AGAAACCCTTGAGCCCGAAG-3′, Scn1ar_rev 5′-CACACTGATTTGACAGCACTTGAA-3′; Nav1.2: Scn2ar_fw 5′-AGGAACGCAAGGACGAAG-3′, Scn2ar_rev 5′-TCTAATGGGGTTGAAGGGAG-3′; Nav1.3: Scn3ar_fw 5′-CGATGCAATTCACCCTGGAAG-3′, Scn3ar_rev 5′-GTGGCGACGCTGAAGTTCTC-3′; Nav1.6: Scn8ar_fw 5′-ATGGTGAGCGGAGATCGAA-3′, Scn8ar_rev 5′-GTGGTCGTGATAGGCTCGTA-3′; Nav1.7: Scn9ar_fw 5′-TCCTTTATTCATAATCCCAGCCTCAC-3′, Scn9ar_rev 5′-GATCGGTTCCGTCTCTCTTTGC-3′; Gapdh: fw 5′-ATCTTCTTGTGCAGTGCCAGCCTCGTC-3′, rev 5′-GAACATGTAGACCATGTAGTTGAGGTCAATGAAGG-3′.

### Western blotting.

After isolation from cell culture medium, exosomes were resuspended in CHAPS Cell Extract Buffer (Cell Signaling Technology, 9852S) supplemented with protease inhibitor cocktail (Thermo Fisher Scientific, 78430), and 4× Laemmli buffer was added to the samples. Following denaturation at 95°C for 5 minutes, 30 μg/lane was loaded on 10% SDS-polyacrylamide gel. Proteins were transferred onto Hybond ECL nitrocellulose membrane (Amersham Biosciences, LC2000) using the semidry system Trans-Blot Turbo Transfer System (Bio-Rad, 1704150) set to 1.3 A and 25 V for 1 hour. Membrane was blocked in 3% BSA (MilliporeSigma) for 1 hour at RT, then incubated with the primary rabbit polyclonal antibody against TNF-α (Abcam, ab6671; 1:500) overnight at 4°C with gentle shaking. Next, three 5-minute washes in TBS–0.1% Tween 20 (MilliporeSigma, P9416) were performed, and goat anti-rabbit HRP-conjugated secondary antibodies (Agilent, P0449; 1:2,000) were added for 1 hour at RT. After washing, the membrane was incubated for 3–5 minutes with Immobilon ECL Ultra Western HRP Substrate (MilliporeSigma, 42029053), and images were acquired with the transilluminator NineAlliance (Uvitec).

### TNF-α quantification.

Exosomes derived from U87 cells and patients were lysed in RIPA cell lysis buffer (NaCl 150 mM, Tris-HCl 50 mM, NP-40 1%, sodium deoxycholate 0.5%, ddH_2_O) containing Protease Inhibitor Cocktail 50× (Thermo Fisher Scientific, 78430). Protein concentration was determined with the Pierce BCA Protein Assay Kit (Thermo Fisher Scientific, 23225), and TNF-α content was quantified using ELISA (Cloud-Clone Corp., SEA133Hu), per standard protocol. The amount of TNF-α per 100 μg of total proteins was determined recording absorbance at 450 nm.

### Electrophysiology.

Hippocampal neurons (DIV 8–12) on coverslips were viewed on an inverted microscope (Olympus, IX70), and neuron identification was made by morphology and later during the recordings in whole cells. Patch electrodes had a 2–5 MΩ resistance and were pulled from borosilicate capillaries (World Precision Instruments) with a PC-10 puller (Narishige); electrodes contained an intracellular solution of (in mM): 145 KCl, 4 MgCl_2_, 1 EGTA, 2 Na_2_-ATP, and 10 HEPES, adjusted to pH 7.2 with KOH. In some experiments to isolate Na^+^ current, there was a partial replacement of KCl by 135 mM of CsCl plus NaCl 5 mM. Electrophysiological recordings were made using a MultiClamp 700B amplifier controlled by Clampex 10.6 via a Digidata 1550B (Molecular Devices); the sample frequency used to acquire data was 10 kHz with low-pass filtering at 2 kHz.

To be sure that neurons were stable, they were held at I = 0 for 3 or 5 minutes. If the analyzed neuron was stable during this time, we started recording spontaneous activity. Then on the same cell, synaptic spontaneous currents (4 minutes, –70 mV) and the activation of voltage-gated currents by step depolarization from a holding potential of –70 mV were recorded in voltage clamp configuration. To check whether cells that did not show spontaneous activity were neurons, a slow hyperpolarizing current was applied until –70 mV, and then a step depolarization protocol was performed by injection of a current by 1.5 seconds with increasing amplitude of 50 pA in each step until APs were triggered. Before current injection, bridge balance and pipette capacitance neutralization were carefully monitored and adjusted throughout experiments by application of low-frequency (50 Hz), low-amplitude (10–40 pA) current steps. AP properties were obtained from the mean phase plot (dV/dt) of 10 APs randomly chosen during the record. Statistical analysis of spike frequency, maximum currents, I-V curves, and conductance plots was made by IGOR pro (WaveMetrics, https://www.wavemetrics.com/).

The extracellular ringer contained (in mM): 140 NaCl, 5 KCl, 2 CaCl_2_, 1 MgCl_2_, 10 HEPES, and 10 glucose, pH 7.4. In some experiments to check the ionic permeability to Na^+^, 2 types of solution were used: the first had 70 mM of NaCl plus 70 mM of NMDG-Cl to decrease the amplitude of Na^+^ currents, and all the other components were the same as the extracellular ringer plus a blocker of Ca^2+^ gated conductance (CdCl_2_ 100 μM); the second was prepared with CdCl_2_ and isosmolar replacement of NaCl by NMDG-Cl. To change solutions, the setup was equipped with an 8-in-1 multibarrel perfusion pencil connected to a ValveLink8.2 pinch valve perfusion system (Automate Scientific). All compounds and chemicals were obtained from MilliporeSigma, unless otherwise stated. All liquid junction potentials for the solutions used were calculated using the pClampex software (Axon Instruments), obtaining values between 1.2 and 3.8 mV.

### Surgical procedures on patients with glioma.

The surgical procedures were performed using cortical and subcortical mapping methods in accordance with the usual surgical technique (99, 100). In addition to direct electrical stimulation, a performance-based resection related to real-time neuropsychological testing was performed in those cases selected for awake surgery (101). Intraoperative neurophysiological monitoring and intraoperative electrocorticography were performed in all cases as previously described (Axon System Eclipse) (102).

### Statistics.

Results are presented as mean ± SD or mean ± SEM, as indicated in the figure legends. Statistical analysis was performed with Prism 7 (GraphPad Software). The 2-tailed unpaired Student’s *t* test was used to compare 2 normally distributed sample groups, and equality of variances was tested through the *F* test; when more than 2 groups were compared, 1-way ANOVA followed by Bonferroni’s post hoc multiple-comparison test was performed, and equality of variances was tested through the Brown-Forsythe and Bartlett’s tests. The Wilcoxon-Mann-Whitney *U* test and Kruskal-Wallis test were used to compare 2 or more non-normally distributed sample groups, respectively. *P* values less than 0.05 were considered significant. No predictive statistical methods were used to predetermine sample sizes; however, we adopted sample sizes (indicated in figure legends) in the same range as those previously reported in the literature for similar experiments. The ROUT method with *Q* = 1% was used to identify outliers. No randomization method was followed to allocate samples to the various experimental groups. Investigators were masked to group allocation and when assessing the outcome of the experiments.

### Study approval.

The use of primary neurons was approved by the Local Veterinary Service (Trieste, Italy), by the SISSA Ethics Committee board, and by the National Ministry of Health. All the procedures were conducted according to the guidelines of the Italian Animal Welfare Act and to the European Union guidelines for animal care (d.1.116/92; 86/609/C.E.).

The protocol for human samples was approved by the local Ethics Committee, Comitato Etico Unico Regionale del Friuli Venezia Giulia (protocol 718345, opinion 196/2014/Em).

### Data availability.

The data that support the findings of this study are available upon reasonable request. The Supporting Data Values file is provided as supplemental material.

## Author contributions

CAST performed and analyzed electrophysiological experiments, discussed the results, and contributed to paper writing. RS performed and analyzed biochemical and immunocytochemical experiments, discussed the results, and contributed to paper writing. FS and CD performed and analyzed biochemical and immunocytochemical experiments. IM, IGR, TI, and DC derived and cultured GSCs and GASCs, discussed the results, and contributed to paper writing. PP characterized the biophysical properties of exosomes. AM supervised electrophysiological experiments and financially supported the project. MS performed neurosurgery and contributed to paper writing. FC supervised the biochemical and immunocytochemical experiments and wrote the paper. VT designed the project, supervised all the experiments, discussed the results, wrote the paper, and financially supported the project. CAST and RS are co–first authors. Because of the prevalence of electrophysiological data, CAST is assigned the first position.

## Supplementary Material

Supplemental data

Unedited blot and gel images

Supporting data values

## Version 1 08/01/2024

In-Press Preview

## Version 2 09/17/2024

Electronic publication
