# Supplementary material for: Exosomal TNF-α mediates voltage-gated Na+ channel 1.6 overexpression and contributes to brain tumor–induced neuronal hyperexcitability
Source: J Clin Invest. 2024 Aug 1;134(18):e166271. doi: 10.1172/JCI166271 (PMC11405049; doi:10.1172/JCI166271)
Supplement: Supplemental data [file jci-134-166271-s196.pdf]

SUPPLEMENTARY DATA

Exosomal TNF-α mediates voltage-gated Na^+ channels 1.6 overexpression and contributes to brain-tumor induced neuronal hyperexcitability

Cesar Adolfo Sanchez Trivino[1], Renza Spelat[1,2]*, Federica Spada[1,3], Camilla D'Angelo[1], Ivana Manini[4], Irene Giulia Rolle[4], Tamara Ius[5], Pietro Parisse[2], Anna Menini[1], Daniela Cesselli[4], Miran Skrap[6], Fabrizia Cesca[3+], Vincent Torre[1,2,7,8+]*

[1]*International School for Advanced Studies (SISSA), via Bonomea 265, Trieste, Italy*

[2]*Institute of Materials (IOM-CNR), Area Science Park, Basovizza, Trieste, Italy*

[3]*Department of Life Sciences, University of Trieste, Italy*

[4]*Università degli studi di Udine, Istituto di Anatomia Patologica, ASUIUD, Italy*

[5]*Neurosurgery Unit, Department of Neurosciences, Santa Maria della Misericordia University Hospital, Udine, Italy*

[6]*SOC Neurochirurgia, Az. Ospedaliera Sanitaria Integrata, Udine, Italy*

[7]*BISS GlioGuard S.r.l., Trieste, Italy*

[8]*Suzhou Institute of Nano-Tech and Nano-Bionics, Chinese Academy of Sciences, Jiangsu, P.R. China*

**equally contributed*

[+]*corresponding authors':*

Vincent Torre: torre@sissa.it

Fabrizia Cesca: fcesca@units.it

Patient Number	Cell type
S479	GASC
S471	GASC
S520	GASC
S58	GASC
S226	GASC
S464	GSC
S496	GSC
S411	GSC

Supplementary Table 1: List of the 8 patients used in this study. Each patient is denoted by the letter S followed by an identification number. The specific cell type used for exosome isolation, GASC (glioma-associated stem cells) or GSC (glioma stem cells), is indicated.

RMP (mV)						
Patient Number	MEAN-Treated	SEM-Treated	neurons (n)	MEAN-Control	SEM-Control	neurons (n)
S479	-43.1	2.6	15	-61.7	4.4	10
S471	-44.2	2.3	6	-66.3	1.4	4
S520	-50.8	1.9	25	-67.6	2.3	14
S58	-51.4	2.6	7	-63.6	1.7	5
S226	-57.7	1.9	7	-63.6	1.7	5
S464	-48.8	3.8	4	-59.2	5.7	5
S496	-42.8	2.1	9	-76.8	7.3	4
S411	-50.2	2.7	9	-75.4	6.0	5
HA	-65.4	3.0	8	-62.0	0.8	4

Supplementary Table 2: Comparison table of the RMP mean values measured in hippocampal neurons under control conditions (neurons treated with exosomes extracted from medium that had not been in contact with cells), in neurons treated with exosomes from 8 patients and from human astrocytes (HA). SEM indicates the standard error of the mean.

AP Frequency (Hz)						
Patient Number	MEAN-Treated	SEM-Treated	neurons (n)	MEAN-Control	SEM-Control	neurons (n)
S479	2.3	0.73	15	0.56	0.14	10
S471	3.19	0.76	6	0.79	0.39	4
S520	2.62	0.7	25	0.43	0.22	14
S58	2.26	0.57	7	0.88	0.31	5
S226	1.58	0.54	7	0.88	0.31	5
S464	2.63	0.4	4	1.18	0.33	5
S496	2.17	0.65	9	1.46	0.54	4
S411	2.09	0.38	9	0.54	0.11	5
HA	0.16	0.08	8	0.13	0.07	4

Supplementary Table 3: Comparison table of the AP Frequency measured as described in Supplementary Table 2.

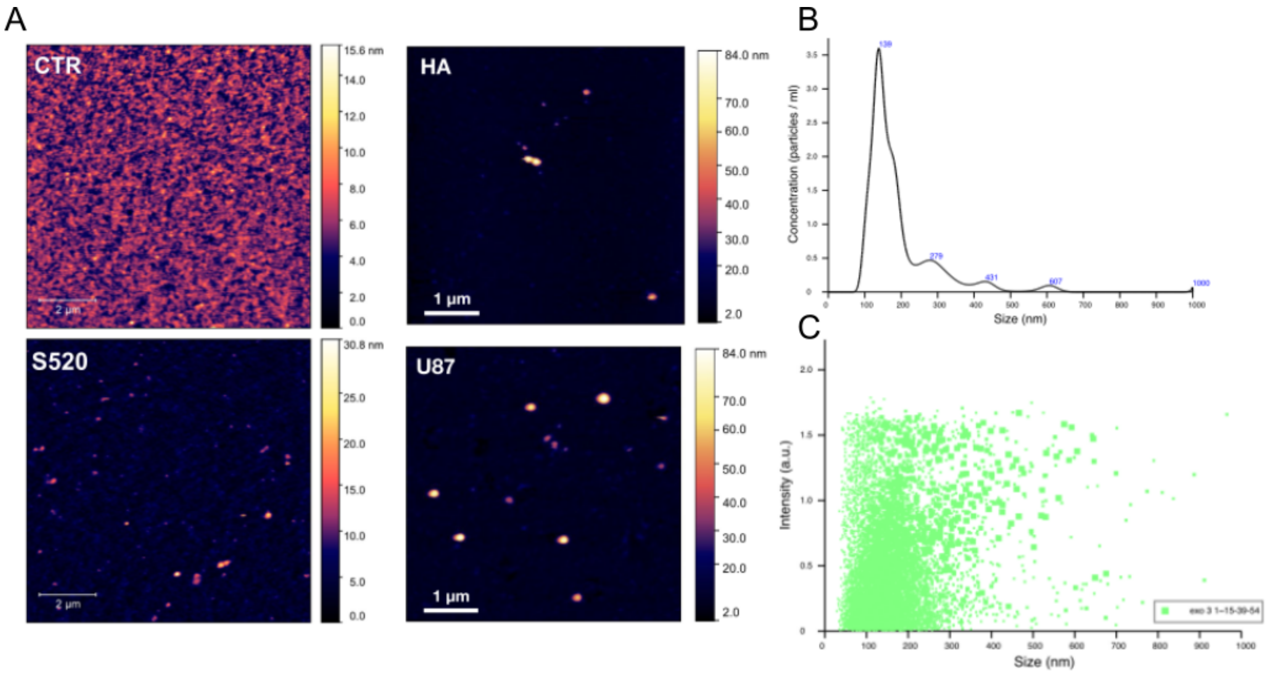

Supplementary Figure 1: Size distribution of exosomes. A. Atomic Force Microscopy analysis of exosomes derived from control (CTR), human astrocytes (HA), patient S520 and U87 cell line (U87), performed in liquid on polyornithine-functionalized supports. Heat legend represents diameter of particles expressed in nm. **B.** Distribution of U87 exosomes based on size (nm) and relative concentration (particles/ml). **C.** Distribution of U87 exosome size (nm) with respect to the relative intensity expressed in arbitrary units (a.u.).

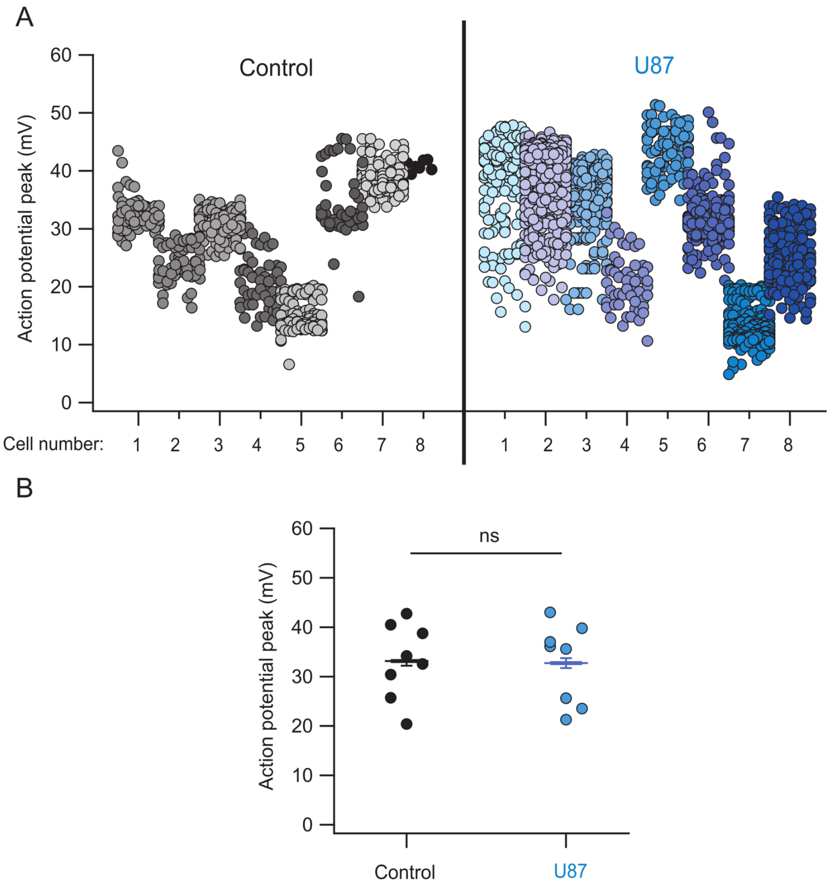

Supplementary Figure 2: Exosome treated neurons and untreated control cells have comparable AP peak. A. Measurements of AP peak for each spike recorded from 8 random control (black and gray colors in the left graph) and U87 exosomes treated neurons (blue and violet colors in the right graph). **B.** Mean of the action potential peak for the cells in A (Mann-Whitney U test, $p>0.05$, $n = 8$).

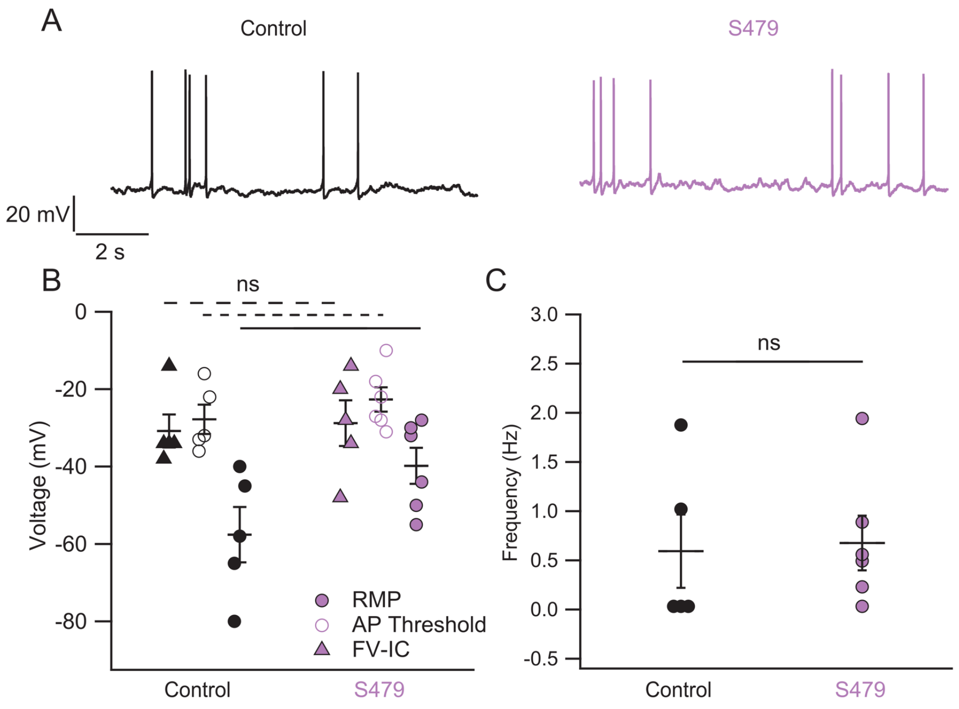

Supplementary Figure 3: Cortical neurons show a high variability of the electrophysiological parameters. A. Representative trace of a control cortical neurons (black trace) and from a cortical neuron treated with exosomes derived from patient #S479 (purple trace). **B.** Comparison of RMP (triangles), AP threshold (empty dots) and FV-IC (colored dots) showing no significant changes after exosome treatment, only RMP show a trend to depolarization similar to that identified in hippocampal neurons. **C.** Spontaneous action potential frequency with a non-significant tendency to increase under exosome treatment. (Mann-Whitney U test, p>0.05 n = 5-6).

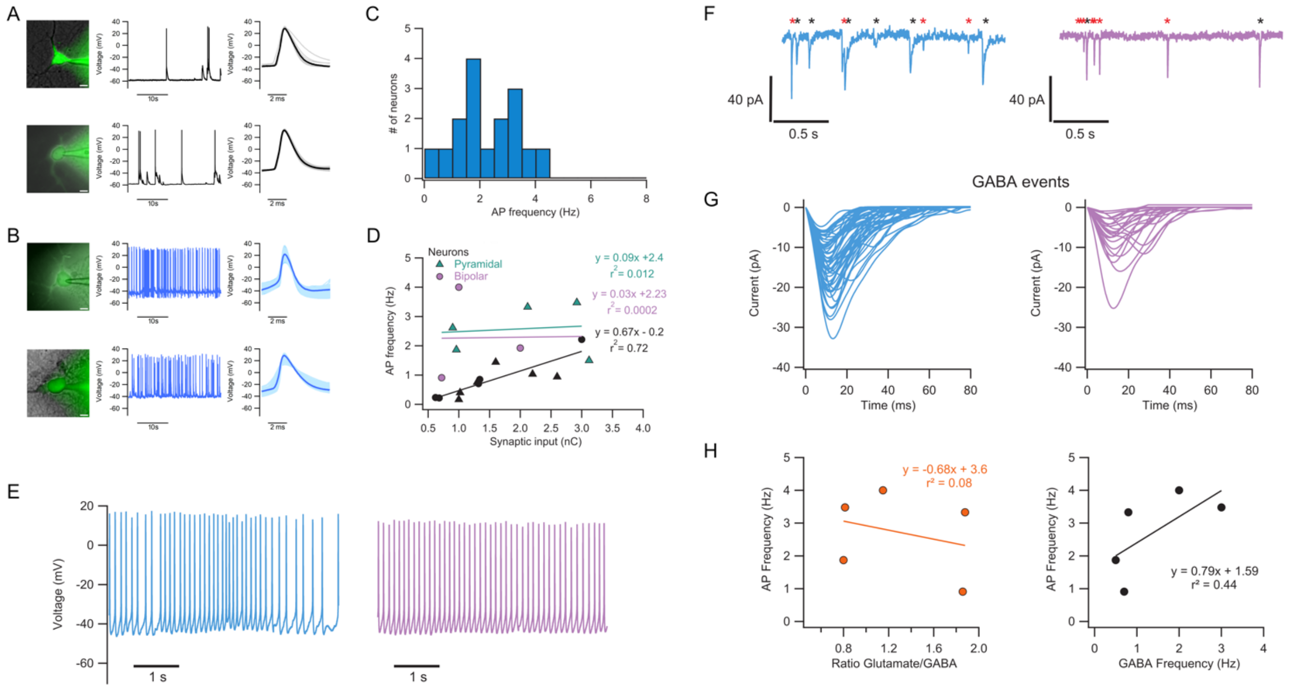

Supplementary Figure 4: Exosomes increase excitability of hippocampal neurons independently of the cellular morphology. A. Representative images of two distinct cellular morphologies recorded in hippocampal cultures under control conditions (black traces). The upper panel shows a pyramidal neuron, while the lower panel shows a bipolar neuron. Both exhibit typical bursting or continuous low-frequency activity (central panel) and action potential shapes with several millivolts of overshoot (right panel). **B.** Similar to the description in (A), this panel illustrates pyramidal and bipolar hippocampal neurons treated with U87-exosomes. These neurons exhibit increased AP frequency and sustained spontaneous activity. In the graphs showing the mean shape of the APs, mean values do not start at RMP around -60 mV because APs sit on a synaptic induced depolarization associated to their bursting behavior. **C.** Distribution of AP frequency in treated neurons, showing two peaks centered at 2 and 3.5 Hz. **D.** AP frequency as a function of synaptic input measured at -80 mV holding potential. While control neurons (black line and symbols) exhibit the expected positive correlation, pyramidal (green) and bipolar (violet) neurons treated with exosomes do not show any correlation, indicating a loss of dependence of firing on synaptic input. **E.** Representative traces in current-clamp of two pyramidal neurons with similar high rates of spontaneous firing. **F.** The same cells as in E recorded in voltage-clamp at -80 mV, displaying spontaneous events of glutamate (red stars; fast decay time events) and GABA (black stars; slow decay time events). **G.** Superposition of GABA events recorded for the same cells as in E and F, showing substantial differences in the GABAergic input frequency, being 1.09 Hz and 0.54 Hz respectively. This indicates that a similar high frequency of APs in two cells is independent of the GABA input. **H.** AP frequency of pyramidal cells as a function of the synaptic ratio of glutamate/GABA (orange) and GABAergic input (black), demonstrating that there is no correlation between a decrease in the synaptic ratio (increase in the GABAergic input) and the spontaneous firing activity of the neurons. Slope and correlation values of the linear regression (r^2) are reported in each panel.

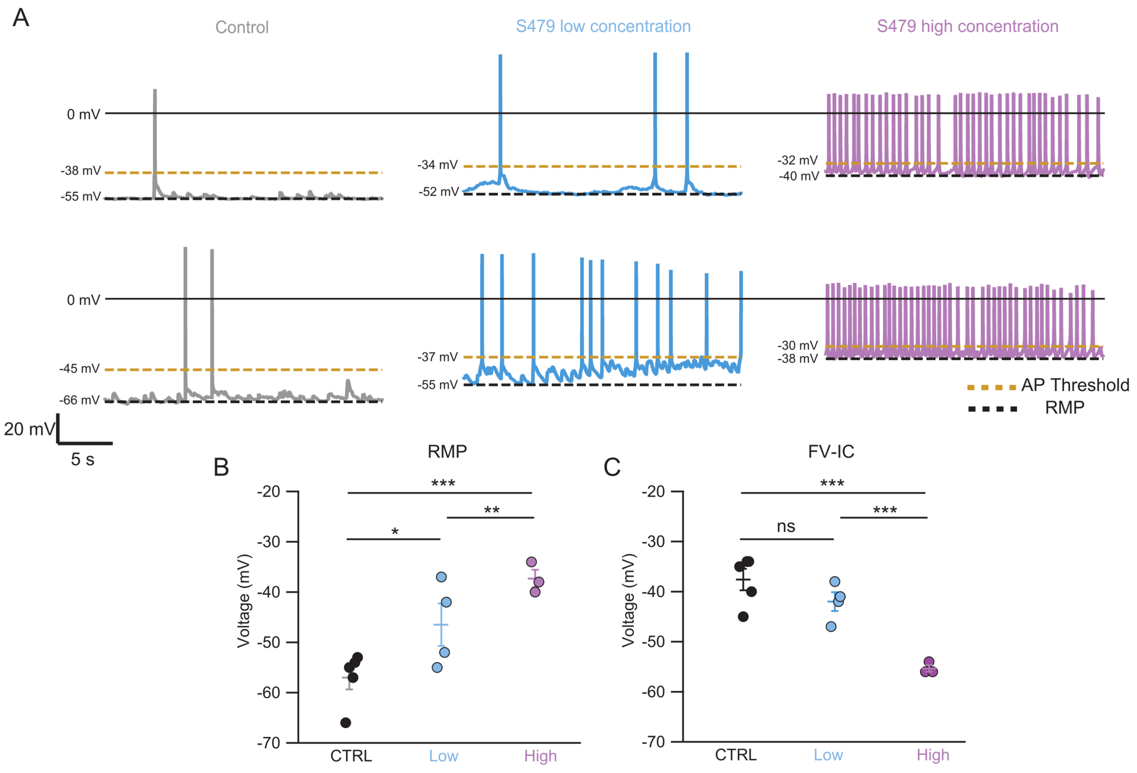

Supplementary Figure 5: Hyperexcitability induced by exosomes is concentration dependent. A. Current clamp recordings of hippocampal neurons in control conditions (gray), incubated with 2.1 x 10^3 particles/cell (blue) and 4.2 x 10^3 particles/cell (purple). Yellow dashed line represents AP threshold, while black dashed line RMP. RMP tends to reach the value of AP threshold trough depolarization in a concentration dependent manner. **B.** RMP values move to depolarized potentials in neurons treated with S479 exosomes. **C.** The voltage clamp depolarized step protocol was applied to identify the FV-IC (amplitude of inward current was considered if it was under 100 pA). Exosome treatment induces a shift of the FV-IC to more negative potentials compared to control neurons Kruskal-Wallis followed by Bonferroni corrected Dunn’s test; *** p<0.001, **p<0.01, *p<0.05. n = 3 to 5.

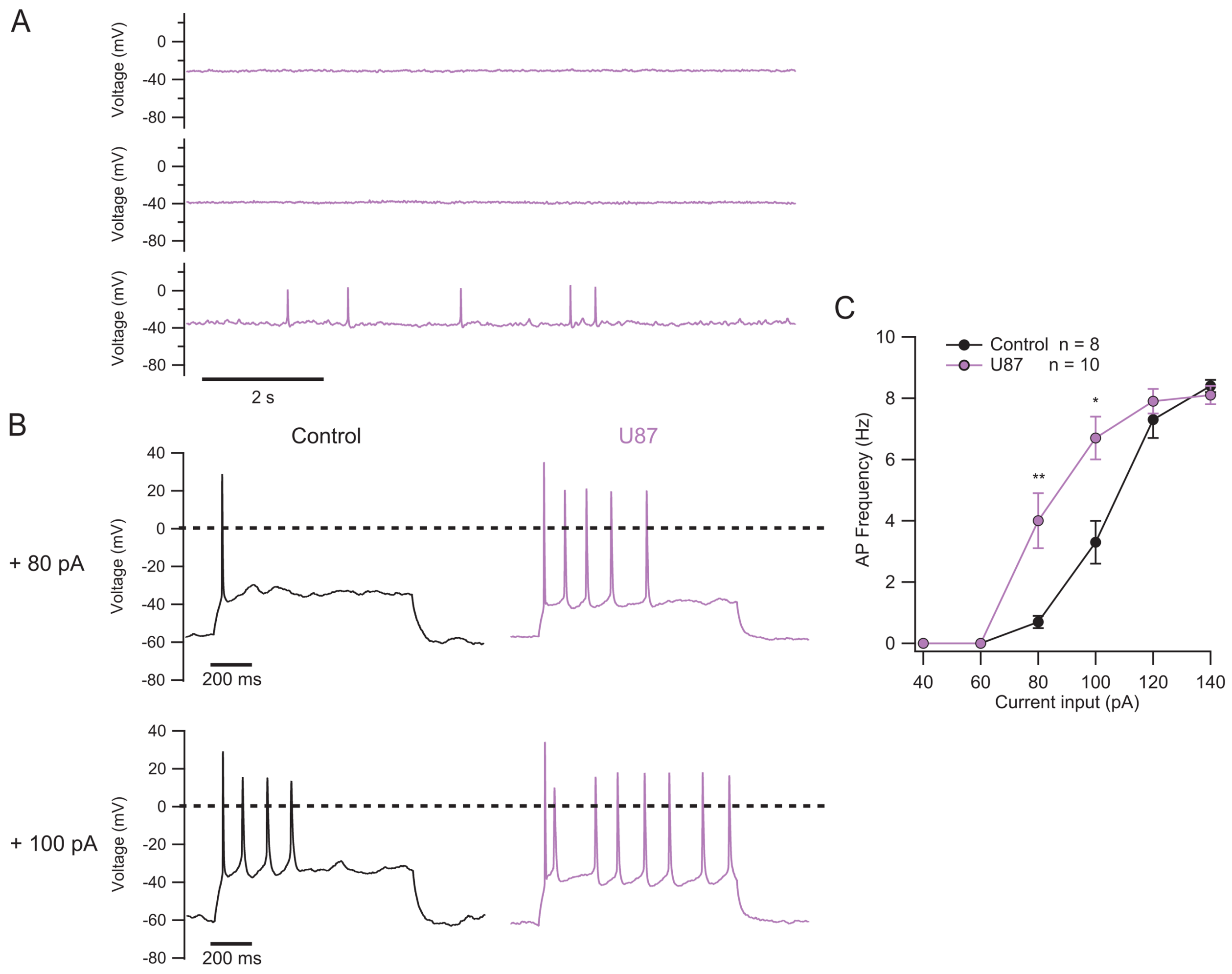

Supplementary Figure 6: Hippocampal neurons treated with U87 exosomes exhibit an inability to generate action potentials. A. Representative traces of neurons recorded in current clamp (I=0) display a highly depolarized resting membrane potential (RMP) ranging from -35 to -40 mV. **B.** A comparison between a control neuron (left, black traces) and the top cell in A, under the injection of a hyperpolarizing current with a holding potential set at -60 mV (right, blue traces). The first and second row recordings were obtained by the injection of a step current of +80 and +100 pA, respectively. **C.** The relationship between the injected current and the firing frequency of action potentials for control neurons (black dots and line) and exosome-treated neurons in A, which exhibit no spontaneous electrical activity (blue dots and line). These data confirm an increase in the excitability of the neurons due to the treatment (Friedman test, **p<0.01).

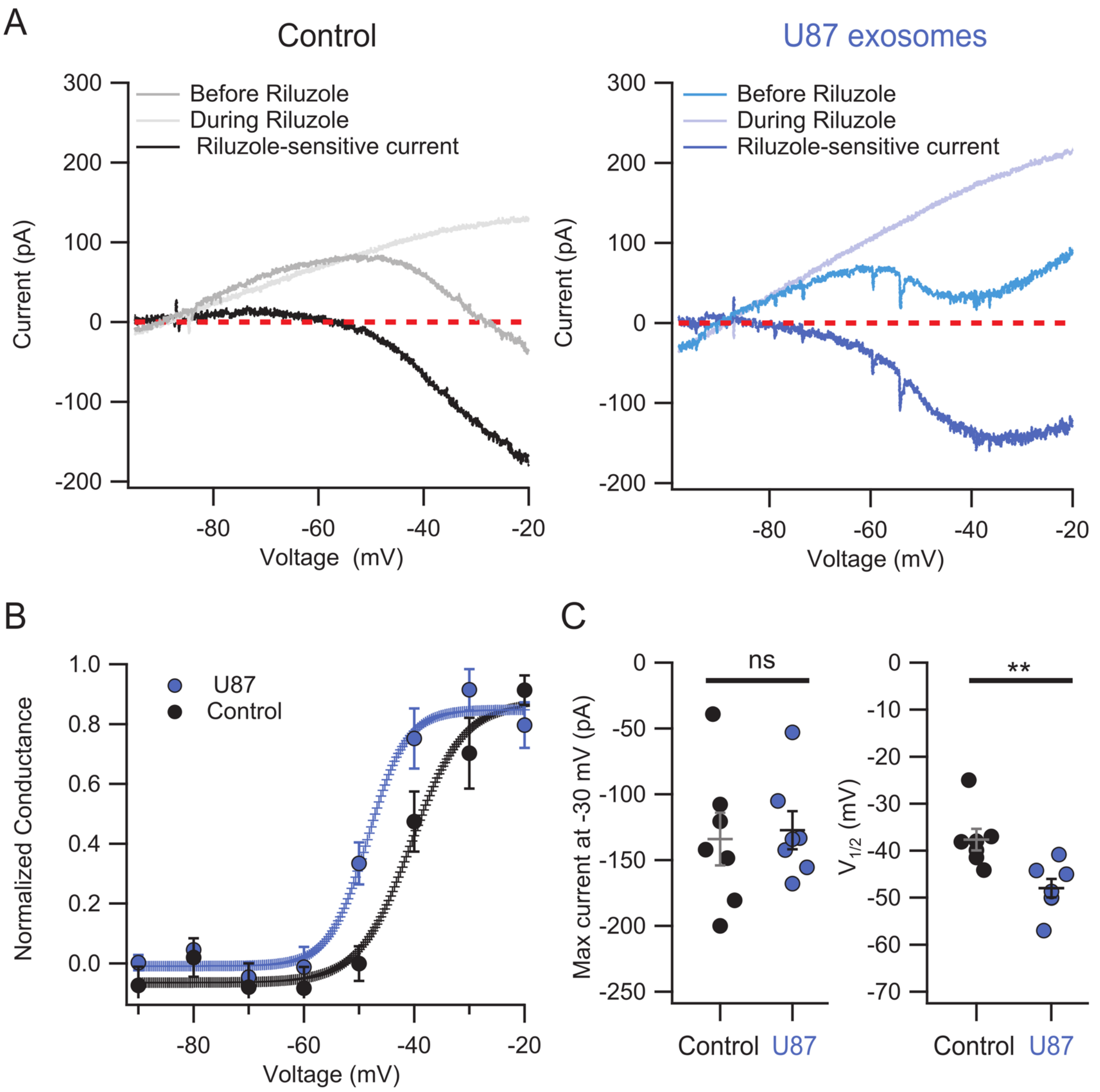

Supplementary Figure 7: Treatment with U87 exosomes induces a negative shift in the activation of the persistence Na⁺ current in hippocampal neurons. A. Application of a slow depolarized ramp to neurons filled with a CsCl-based intracellular solution and 5 mM of NaCl, as shown in experiments in Fig. 5, revealed a persistent Na^+ inward current sensitive to riluzole (10 μM). The dark traces (black = control and blue = treated neurons in all panels) represent the result of subtracting currents evoked during riluzole treatment from those before treatment. **B.** Normalized conductance under both conditions was fitted to a Boltzmann equation, showing an early activation of the persistent Na^+ current in neurons treated with U87 exosomes compared to control. **C.** Magnitude of currents at -30 mV, where the persistence Na^+ conductance is maximal, do not show statistically significant differences (left graph, mean control=-134±20 pA and mean treated neurons=-127.3±14.4 pA). On the contrary, $V_{1/2}$ for activation of persistence current shifts to negative potentials (right graph, mean control=-37.6±2.3 mV and mean treated neurons=-48±2 mV). Statistical analysis was performed with Mann-Whitney U test, **p<0.01, n = 7.

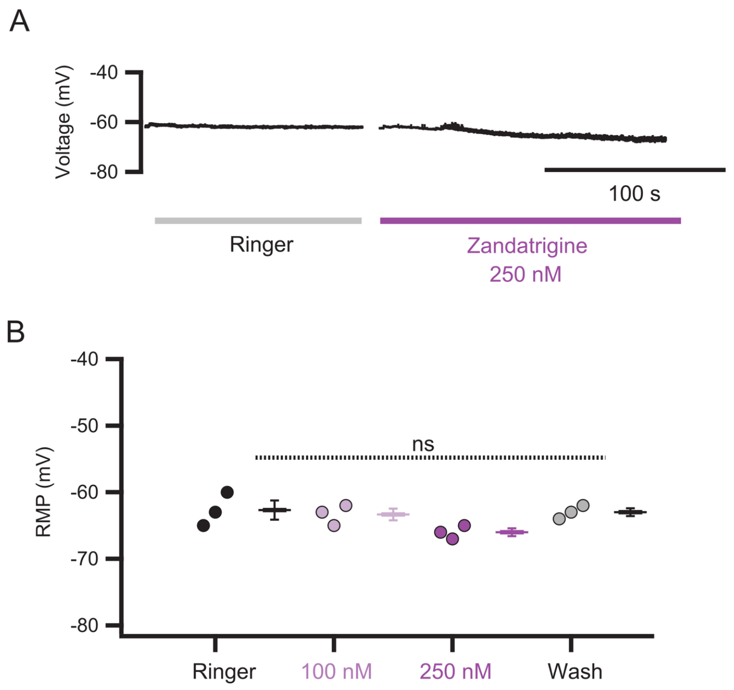

Supplementary Figure 8: RMP of control hippocampal neurons treated with the Nav1.6 inhibitor Zandatrigine. A. Representative trace of control hippocampal neurons during perfusion of Ringer's solution and the transition to acute application of Zandatrigine 250 nM. **B.** RMP measurements in 3 control neurons (black circles, mean=-63+/-1.5 mV) showing that Zandatrigine treatment did not produce statistically significant changes both at low (pale violet dots; mean=-63.5+/-0.88 mV) and high (dark violet dots; mean=-66+/-0.6 mV) concentrations, indicating that Nav1.6 is not involved in the determination of the RMP under control conditions (Kruskal-Wallis test, $p>0.05$, $n=3$).

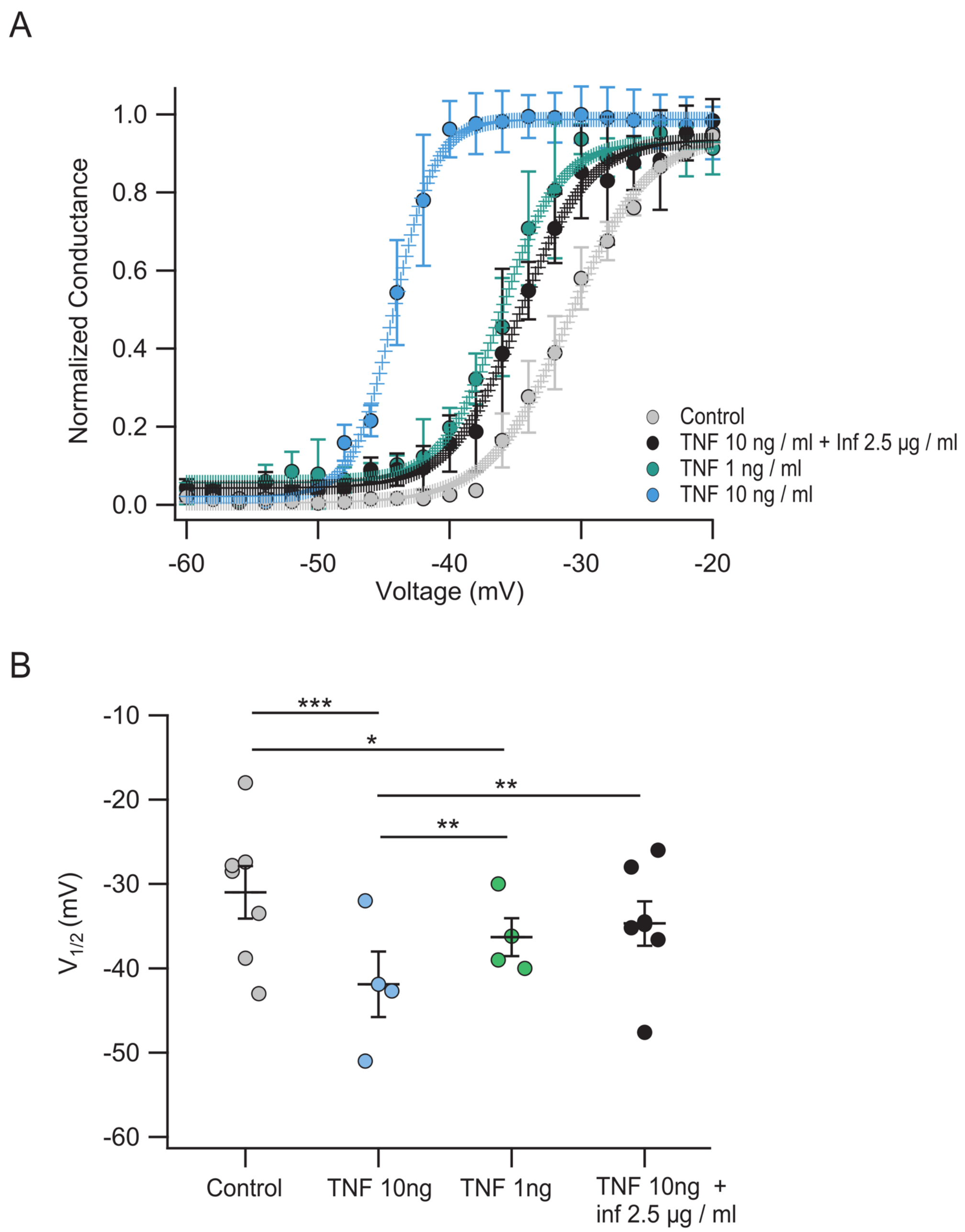

Supplementary Figure 9: TNF-α shifts the activation curve of the Na^{+} inward current towards more negative voltages. A. Activation curve and **B.** corresponding $V_{1/2}$ values. In this series of experiments, the $V_{1/2}$ of activation in controls neurons was -31±3 mV (n=7, gray curve and dots), for neurons treated with 10 ng/ml of TNF-α was -43.9±2.4 mV (n=4, blue curve and dots), values similar to that observed in neurons treated with glioma exosomes. This effect exhibited concentration dependency, as TNF-α at 1 ng/ml shifted the activation curve to a less negative value (-36±2.2 mV, n=4, green curve and dots). Preincubation with Infliximab (n=7, black curve and dots) successfully blocked the effect of 10 ng/ml of TNF-α (-34.6±2.6 mV), and this difference was statistically significant (Kruskal-Wallis followed by Bonferroni corrected Dunn's test ***$p<0.001$).The treatments with TNF-α 1 ng/ml and TNF-α 10 ng/ml + 2.5 ug/ml of Infliximab induced a significant shift towards more negative potentials compared to the control (Dunn's test *$p<0.05$), and treatment with TNF-α 10 ng/ml was statistically different compared to all the other experimental groups (Dunn's test ***$p<0.001$).

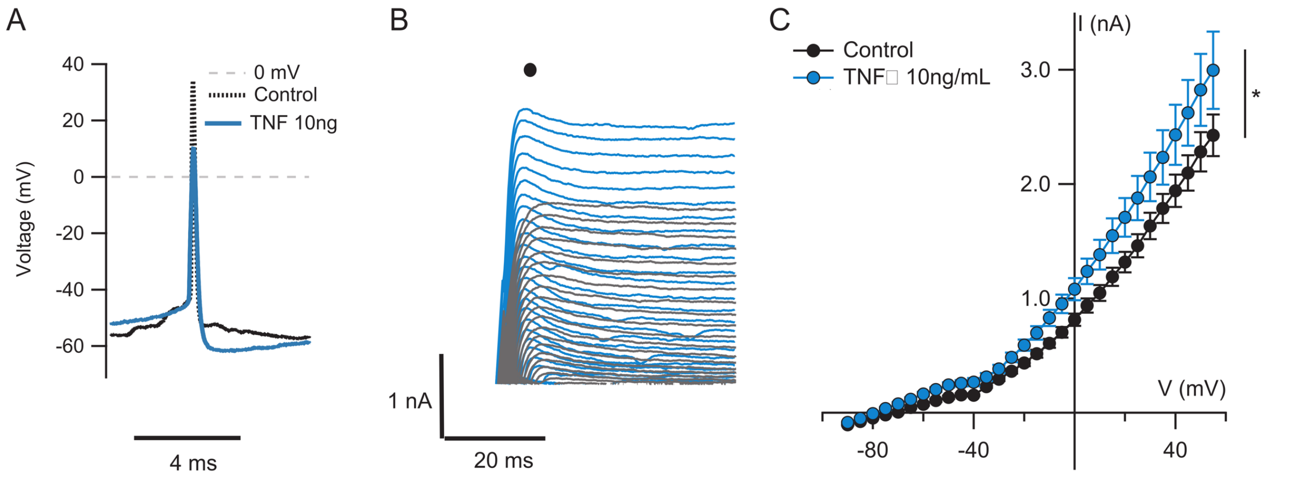

Supplementary Figure 10: TNF-α treatment increases the amplitude of the type-A outward current in hippocampal neurons. A. Action potentials in TNF-α treated neurons displayed a noticeable undershoot (blue trace) in comparison to control neurons. **B.** Representative traces evoked by voltage clamp depolarizing steps (Δ5 mV) keeping the cell at a holding potential of -90 mV: TNF-α-treated neurons (blue traces) showed an increase in the amplitude of the transient outward current compared to control neurons (black traces). **C.** The IV curve was calculated from the positive peak current in B (black dot), revealing an increase in the amplitude of the current under TNF-α treatment. The active current at the depolarized step to +40 mV in control neurons was 1941.8±141.6 pA, a lower value than the 2430.3 ± 262.6 pA observed in treated neurons. Kruskal-Wallis; *p<0.05; n=10 controls; 17= treated.
