# Supplementary figures and images for: Exosomal TNF-α mediates voltage-gated Na+ channel 1.6 overexpression and contributes to brain tumor–induced neuronal hyperexcitability
Source: J Clin Invest. 2024 Aug 1;134(18):e166271. doi: 10.1172/JCI166271 (PMC11405049; doi:10.1172/JCI166271)

Trivino et al, 2024

TNF- $\alpha$  blot in Fig. 6A (original image)

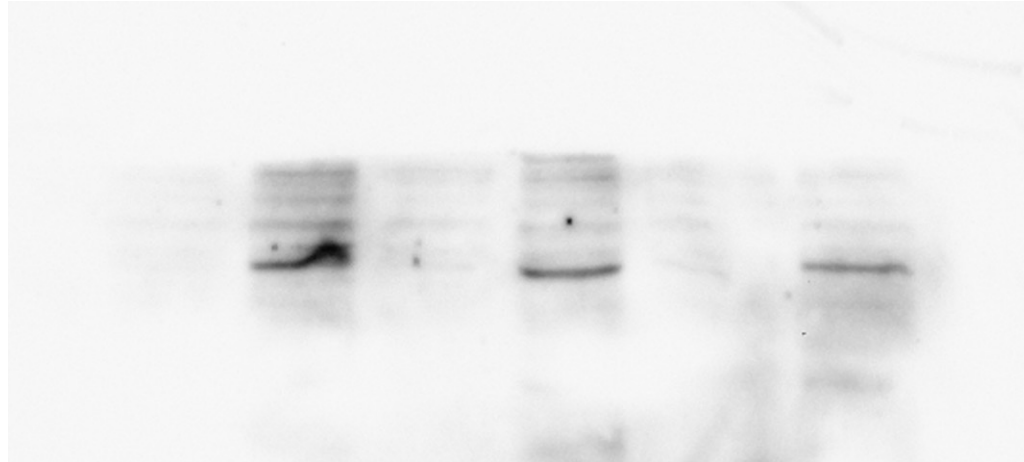

ALIX blot in Fig. 6A (original image)

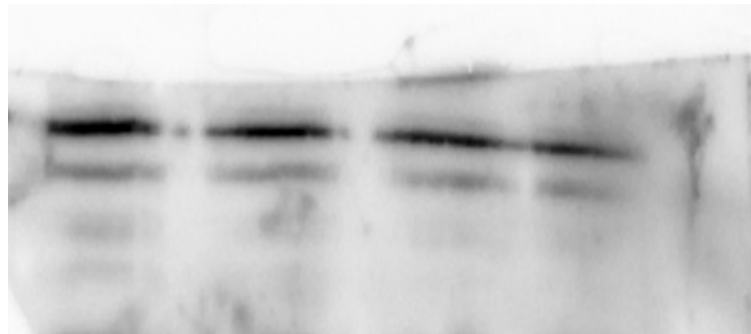

Supplement: Unedited blot and gel images [file jci-134-166271-s197.pdf]
